# Data harmonization processes of cancer data into the observational medical outcomes partnership common data model

**DOI:** 10.1038/s41598-026-53570-9

**Published:** 2026-05-22

**Authors:** Ifani Pinto Nada, Stefano Bonacina

**Affiliations:** Department of Learning, Informatics, Management and Ethics, Health Informatics Centre, Karolinska Institutet, Stockholm, Sweden

**Keywords:** Cancer, Computational biology and bioinformatics, Oncology

## Abstract

Cancer data is inherently complex and heterogeneous, originating from diverse sources with differing formats, terminologies, and structures, leading to significant interoperability challenges. The Observational Medical Outcomes Partnership (OMOP) Common Data Model (CDM), provided by Observational Health Data Sciences and Informatics (OHDSI) initiative, has been adopted as a standardized framework to mitigate data fragmentation and enhance evidence generation. However, harmonizing cancer data into OMOP CDM remains challenging due to granular data, unstructured formats, and lack of cancer-specific harmonization approaches. This study develops a generic harmonization process for integrating cancer data into the OMOP CDM by examining existing methodologies and identifying patterns and challenges. Following the Design Science Research Methodology (DSRM), the process was informed by literature and refined through expert feedback. The proposed process consists of five steps: Initiation, Requirement Analysis, Design Planning, Technical Implementation, and Maintenance. Each step incorporates cancer-specific considerations. It addresses challenges including source data quality and complexity, mapping issues, and maintenance, supporting oncology research and evolving technologies.

**Supplementary Information:**

The online version contains supplementary material available at 10.1038/s41598-026-53570-9.

## Introduction

Cancer data is inherently complex and heterogeneous, requiring detailed characterization of tumor histology, biomarkers, disease progression, and multimodal treatment pathways^1–6^. These data originate from diverse sources, including Electronic Health Records (EHRs), insurance claims, and cancer registries, each using different formats, terminologies, and structures, leading to significant interoperability challenges^1^.

To reduce data fragmentation and enhance Real-World Evidence (RWE) generation, researchers have adopted Observational Medical Outcomes Partnership (OMOP) Common Data Model (CDM) as a standardized framework for cancer data integration^7–14^. However, harmonizing cancer data into OMOP CDM remains highly challenging due to three key factors:

1. **Granular data requirements**
  Unlike other conditions, cancer requires highly detailed characterization, including tumor histology, biomarkers, genomic variations, disease staging, complex treatment pathways, and longitudinal disease progression. Capturing this complexity often requires the abstraction of low-level clinical events into higher-level representations of patient episodes to reflect the full treatment and disease trajectory^4,5^.
2. **Unstructured data challenges**
  Critical cancer-related information, such as cancer staging and biomarker data, is often stored in free-text clinical notes, requiring additional manual curation and extraction steps using advanced Natural Language Processing (NLP)^1,15–20^.
3. **Lack of standardized**,** cancer-specific harmonization approaches**
  Existing frameworks such as Observational Health Data Sciences and Informatics (OHDSI)’s four best practices^21^ and Henke et al.’s nine-step^22^ harmonization process provide general guidance, but they do not fully address the unique complexities of oncology data integration, particularly for handling unstructured text and granular oncology-specific mappings.

These limitations often lead researchers to develop ad hoc, dataset-specific harmonization strategies, which reduce reproducibility and scalability^7^. To bridge this gap, this research aims to develop a generic harmonization process tailored specifically for cancer data integration into OMOP CDM. The study has three objectives:

1. To analyze existing cancer data harmonization methodologies into the OMOP CDM and identify similarities and differences.
2. To identify challenges associated with cancer data harmonization into the OMOP CDM.
3. To design a generic harmonization process for cancer data into the OMOP CDM.

By standardizing this process, it seeks to enhance research reproducibility and accelerate oncology-related discoveries. In this study, the term research refers to the secondary use of harmonized cancer data within the OMOP CDM for observational analytics, real-world evidence generation, and federated analyses^23^. This definition emphasizes data-driven observational investigations rather than interventional clinical trials. This work contributes to broader health informatics initiatives by establishing a standardized approach for cancer data harmonization within OMOP CDM, enabling more effective RWE generation. The study aims to develop a generic harmonization process for integrating cancer data into the OMOP CDM by examining existing methodologies and identifying patterns and challenges specific to cancer data to facilitate robust oncology research.

## Results

### Literature review

**Fig. 1:**
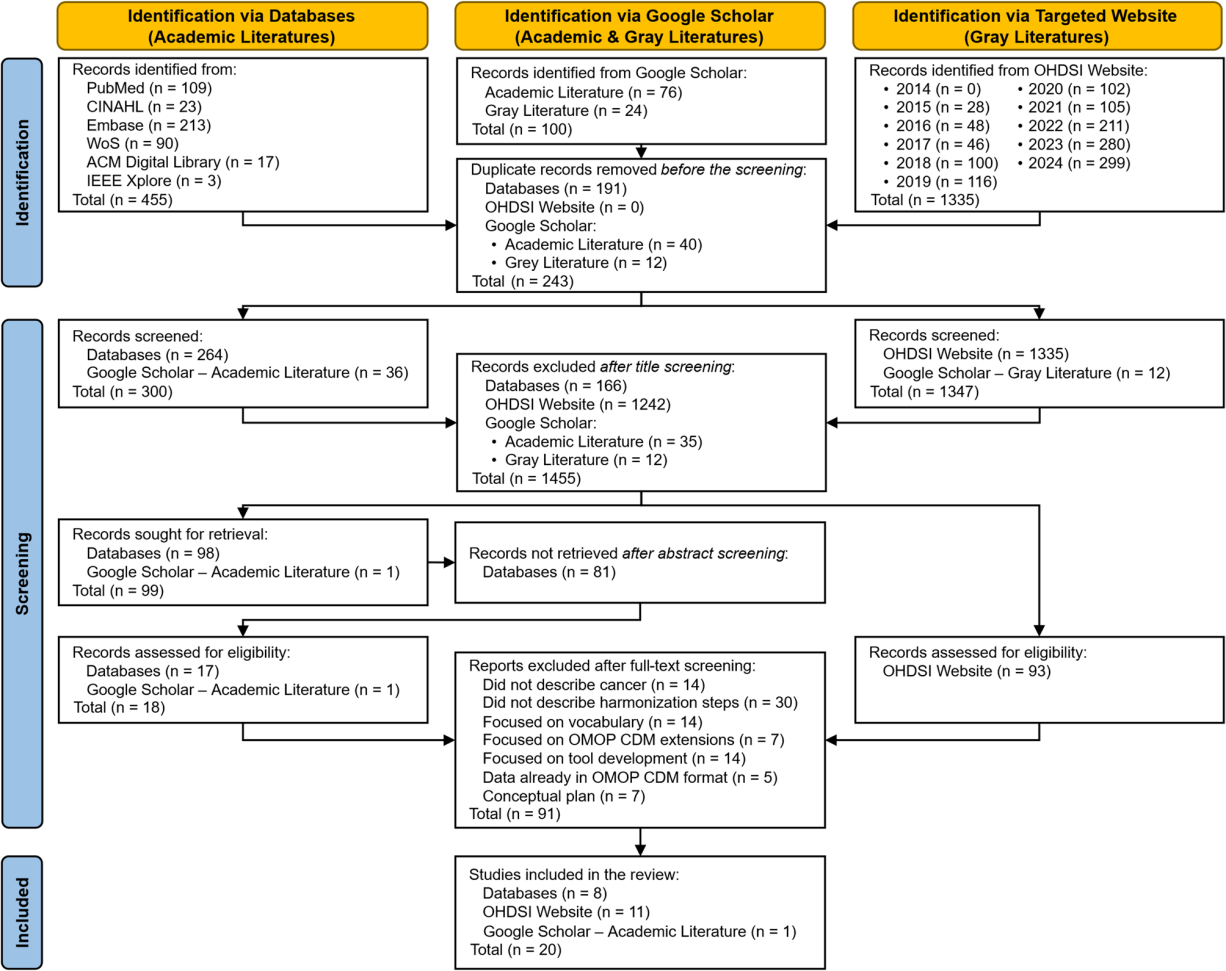
PRISMA flow diagram. Article inclusion process from identification, screening, and selection through six academic databases for academic literature, Google Scholar for both academic and gray literature, and targeted website for gray literature.

The initial search across six academic databases retrieved 455 articles, while 1,335 Gy literature sources were identified in the OHDSI website’s symposium collaborator showcase section. In addition, the first 100 articles based on the relevancy from Google Scholar containing both academic and gray literatures were obtained. After screening, 9 academic articles (45%) and 11 Gy literatures (55%) met the inclusion criteria, totaling 20 reviewed articles. The article selection process is illustrated in the Preferred Reporting Items for Systematic reviews and Meta-Analyses (PRISMA) flow diagram (Fig. 1).

The reviewed articles were published between 2016 and 2024, with the majority published from 2022 to 2024. A detailed breakdown of publication years and their characteristics is available in Table 1. While only 20 articles were included in total, some articles addressed multiple cancer types, data source types, or OHDSI tools. As a result, the summed counts for certain categories in the table may exceed 20.

**Table 1:** Included articles characteristics.

Category	Total (%)
**Literature Type**	**20 (100%)**
Academic Literature	9^5,24–31^ (45%)
Gray Literature	11^32–42^ (55%)
**Published Year**	**20 (100%)**
2016 2018 2019 2020 2022 2023 2024	1^32^ (5%) 2^25,26^ (10%) 2^5,33^ (10%) 1^27^ (5%) 4^24,28,34,35^ (20%) 6^29,30,36–39^ (30%) 4^31,40–42^ (20%)
**Intended Use Case**	**20 (100%)**
Clinical & translational oncology research	6^27,33,35,38,39,42^ (30%)
Oncology population health studies & analytics	14^5,24–26,28–32,34,36,37,40,41^ (70%)
**Total Cancer Types Discussed in Article**	**20 (100%)**
General Cancer	9^5,28–30,34,36,39–41^ (45%)
Multiple Cancer Types	1^37^ (5%)
Single Cancer Type	10^24–27,31–33,35,38,42^ (50%)
**Cancer Types**	**27 (100%)**
***Blood & Lymph System*** B-cell Cancer Leukemia	**3 (11.11%)** 1^32^ 2^27,38^
***Breast*** Breast Cancer	**2 (7.41%)** 2^24,37^
***Digestive System*** Colorectal Cancer	**6 (22.22%)** 4^26,33,35,37^
Intrahepatic Cholangiocarcinoma	1^25^
Upper Gastrointestinal	1^37^
***General***	**9**^5,28–30,34,36,39–41^ **(33.33%)**
***Head and Neck*** Head & Neck Cancer	**1 (3.70%)** 1^37^
***Lung*** Lung Cancer	**2 (7.41%)** 2^37,42^
***Reproductive System*** Gynecological Cancer Prostate Cancer	**2 (7.41%)** 1^37^ 1^31^
***Skin*** Skin Cancer	**1 (3.70%)** 1^37^
***Urinary System*** Urological Cancer	**1 (3.70%)** 1^37^
**Total Data Source Types Discussed in Article**	**20 (100%)**
Single Data Source	10^24,25,27,29,33,34,39–42^ (50%)
Multiple Data Sources	10^5,26,28,30–32,35–38^ (50%)
**Data Source Types**	**33 (100%)**
Biobank Cancer Registry Claims Data EHRs Patient Reported Outcomes (PROs) Other Health Databases	2^27,28^ (6.06%) 10^5,24,28,29,32,34–37,41^ (30.3%) 4^28,30,32,36^ (12.12%) 9^5,25,26,28,30,33,39,40,42^ (27.27%) 2^28,31^ (6.06%) 6^28,30,31,35,36,38^ (18.18%)
**OHDSI Tools & Algorithms**	**51 (100%)**
Achilles ARES ARTEMIS Athena ATLAS DQD KOIOS OncoRegimenFinder Rabbit In A Hat Usagi White Rabbit	6^26,28–30,35,38^ (11.76%) 1^37^ (1.96%) 2^39,40^ (3.92%) 8^25–31,42^ (15.69%) 7^28,31,33,35–37,42^ (13.73%) 7^28,30,31,35,36,38,40^ (13.73%) 1^40^ (1.96%) 2^24,29^ (3.92%) 4^26,35,38,39^ (7.84%) 7^26,28–31,38,42^ (13.73%) 6^26,28,31,35,38,39^ (11.76%)
**Data Origin by Region and Country**	**20 (100%)**
***Americas*** U.S.	**4**^5,27,32,39^ **(20%)**
***Europe*** Denmark Estonia Finland Germany Greece Ireland Netherlands UK	**14 (70%)** 1^35^ 1^30^ 2^36,40^ 4^24,26,29,31^ 1^38^ 1^37^ 1^34^ 3^28,41,42^
***Asia*** China South Korea	**2 (10%)** 1^25^ 1^33^

### Intended use case in the literature

Two dominant intended use cases for harmonization were identified across the reviewed articles. Approximately 30% of the studies^27,33,35,38,39,42^ were interpreted as supporting observational and real-world data–driven activities within clinical and translational oncology research, including improving cancer care through personalized or precision medicine approaches at the patient level. The remaining 70% of articles^5,24–26,28–32,34,36,37,40,41^ were interpreted as supporting oncology population health studies and analytics, including disease surveillance, survival analysis, evaluation of treatment and outcome trends, expansion and integration of cancer registries, and improvements in standardized reporting. These intended use cases were inferred from contextual cues when explicit statements of purpose were absent.

### Cancer types covered in the literature

Half of the reviewed articles^24–27,31–33,35,38,42^(50%) focused on a single cancer type, while 45% discussed general cancer^5,28–30,34,36,39–41^, and only one article^37^ addressed multiple cancer types. Among the specific cancers, digestive system cancers were the most frequently discussed, appearing in 22.22% of the articles^25,26,33,35,37^. The categorization of cancer types follows the classification by body system^43^.

### Data sources used in the literature

Half of the studies (50%) relied on a single data source^24,25,27,29,33,34,39–42^, while the other half used multiple sources^5,26,28,30–32,35–38^. Among these, cancer registries were the most frequently used (30.3%), followed by electronic health records (EHRs) (27.27%). Other health databases included death registries^36^, national health databases^35^, prescription databases^30^, and research databases^28,31,38^. Notably, none of the reviewed articles mentioned the use of standardized data models such as openEHR or Health Level Seven (HL7) Fast Healthcare Interoperability Resources (FHIR).

### Use of OHDSI tools

The OHDSI community provides a suite of tools and algorithms to support data harmonization, mapping, and quality assessment within the OMOP CDM. These tools and algorithms (Table 2) facilitate the semantic and structural mapping, Extract-Transform-Load (ETL) process, and data quality evaluation, ensuring standardization across datasets. 80% of the reviewed studies referenced OHDSI tools, with Athena being the most commonly used (15.69%), followed by Atlas, DQD, and Usagi (each 13.73%). Other OHDSI tools, such as KOIOS and OncoRegimenFinder, were used to a lesser extent.

**Table 2:** OHDSI tools and algorithms for data harmonization.

OHDSI ools	Function
White Rabbit	Helps in understanding the source data, offering detailed insights into the structure and content of the data, which is essential for ETL design^21,44^. The tool generates a comprehensive scan report in an Excel file, includes an overview tab of fields, data types, maximum field length, row counts, and the frequency of empty fields.
Rabbit In A Hat	Facilitates defining and designing the mapping logic required to transform source data into the OMOP CDM^21^. This tool leverages the White Rabbit scan-generated Excel report to create a mapping between source data tables and fields and the corresponding OMOP CDM structures through a user-friendly graphical interface^21,44^.
Athena	Enables users to search OMOP Standardized Vocabularies and download selected vocabulary tables in a zip file for use with the OMOP CDM^44^.
Usagi	Design for manual source code mapping of non-standard vocabulary to the OMOP CDM Standardized Vocabularies^21,44^. The tool also considers synonyms and related names in the vocabulary to enhance mapping precision^21,44^. However, all automated suggestions require manual review to ensure accuracy^21^.
KOIOS	Facilitates the mapping of somatic genomic variants, including genes, genomes, transcripts, and proteins, to the corresponding OMOP Genomic Vocabulary^45^.
OncoRegimenFinder	Maps administered drugs, classified using Anatomical Therapeutic Chemical (ATC) terminology, within 30-day time window to corresponding regimens in the HemOnc vocabulary^46^.
ARTEMIS	Identifies chemotherapy regimens from longitudinal EHR drug records and aligns them with HemOnc-defined regimen as reference^47,48^.
Achilles	Provides descriptive statistics and visual summaries of an OMOP CDM instance by running characterization analyses on the database^44^.
Data Quality Dashboard (DQD)	Check the qualitative data analysis for the plausibility, conformity, and completeness of the source data^49^. Systematically examines each table and field, quantifying records that do not meet specified specifications^44^.
ATLAS	Facilitates quantitative data analysis that compare record counts from source data and OMOP CDM^22^, ensuring data consistency before and after harmonization.
A Research Exploration System (ARES)	A combination of reports and visualizations derived from Achilles and Data Quality Dashboard (DQD) outputs provides detailed characterization of data sources and highlights potential data quality issues effectively^50^.

### Geographic origin of data in the literature

Seventy percent of the reviewed studies used clinical data from the European region^24,26,28–31,34–38,40–42^, while only four and two studies used clinical data from Americas^5,27,32,39^ and Asia^25,33^, respectively. Notably, no data from Sweden was found in the reviewed articles. Regional classification is based on geographic groupings^51^.

### Challenges in cancer data harmonization

Table 3 summarizes the key challenges identified during the harmonization process.

**Table 3:** Challenges associated during cancer data harmonization.

Themes	Subthemes	Total (*n* = 20)
Source Data Quality & Complexities	Structural differences require extensive reformatting.	7^26,28,29,32,34,37,41^ (35%)
	Missing details necessitates manual curation or lead to information loss.	9^27–31,35,38,39,42^ (45%)
	Integrating multiple sources, unstructured data, and PROs requires significant transformation.	7^24,28,29,31,32,37,38^ (35%)
Mapping Issues	Unmapped codes and semantic loss due to incomplete or incorrect mappings.	13^24–32,34,36,38,39^ (65%)
	OMOP vocabularies lack granularity to map certain concepts and represent patient pathways and episodes of care.	8^24–27,29,30,34,41^ (40%)
	Difficulties in mapping free text, PROs, imaging, and genomic data as well as abstracting to derive information, require extensive preprocessing	12^24–26,28–33,35,39,40^ (60%)
	Manual mapping is time-consuming and requires collaboration among experts.	3^26,29,34^ (15%)
Maintenance Issues	Rapid advancements in OMOP CDM require regular maintenance as it affects the harmonization process.	3^24,26,30^ (15%)

### Cancer data harmonization process pattern

Based on the identified patterns from literature review, the key steps in the cancer harmonization process are categorized as follows in Table 4.

**Table 4:** Key steps in the cancer harmonization process into OMOP CDM.

Themes	Subthemes	Total (*n* = 20)
Initiation	Define the intended use case for harmonization, including inclusion and exclusion criteria.	20^5,24–42^ (100%)
	Familiarize and set up the OHDSI ecosystem, including OMOP CDM, standardized vocabularies, and necessary tools and other related infrastructures.	10^25–27,29,31,35–38,41^ (50%)
	Assemble an interdisciplinary team to guide the process.	9^26,27,29–31,35,38,40,42^ (45%)
Requirement Analysis	Analyze data information, vocabulary coverage, and data structure.	18^24–35,37–42^ (90%)
Design Plan	Develop an ETL strategy, defining transformation rules and mapping strategies.	16^5,24–26,28,29,31–37,39–41^ (80%)
	Conduct semantic and structural mapping	19^5,24–32,34–42^ (95%)
	Evaluate ETL strategy.	6^26,28,31,34,36,40^ (30%)
Technical Implementation	Perform data preprocessing (e.g., cleaning, deduplication, de-identification, handling missing values, etc.) and evaluate it.	13^24–26,28–33,35,38–40^ (65%)
	Extract-Transform-Load data into OMOP CDM	20^5,24–42^ (100%)
	Perform quality control and assurance of overall transformation.	18^5,24–33,35–40,42^ (90%)
Maintenance	Regularly update OMOP CDM, vocabularies and OHDSI tools to accommodate evolving data requirements.	4^24,26,29,30^ (20%)

### Evaluation

The proposed process was iteratively refined through three rounds of feedback from academic (author SB) and domain experts. The preliminary evaluation by SB assessed the general overview of the proposed harmonization process that led to the initial refinements. The second evaluation involved domain experts in cancer data harmonization, identified through relevant articles cited in this research and recommendations. Of the 102 individuals contacted, 10 completed interviews and eight completed the questionnaire, conducted concurrently as complementary methods. Overlap between participants in the two components could not be determined. Participant characteristics are presented in Supplementary Fig. 1 and Supplementary Tables 1–2; interview and questionnaire participants are referred to as IP and QP, respectively.

The final evaluation was again conducted by SB, who provided theoretical and methodological feedback. While the process was deemed well explained, several concerns were raised to enhance its rigor and applicability.

### Evaluation criteria assessment

Table 5 shows the frequency distribution and descriptive statistics (total and mean scores) from the questionnaire evaluation of the proposed harmonization process. The evaluation was based on four criteria: ease of use, completeness, efficiency, and generality. Participants rated these criteria on 5-point Likert scale (1 = strongly disagree to 5 = strongly agree), with an “unable to answer” option if not applicable.

**Table 5:** Challenges associated during cancer data harmonization.

Criteria assessment	Number of responses			Total score	Mean
	Unable to answer	Agree	Strongly agree		
Ease of use	1	4	3	31	4.43
Completeness	1	4	3	31	4.43
Efficiency	1	2	5	33	4.71
Generality	1	5	2	30	4.29

One participant selected “unable to answer” for all items; these responses were excluded as they did not constitute a substantive evaluation. Therefore, only seven valid responses were included in the final analysis. “Agree” and “Strongly Agree” were scored as 4 and 5, respectively.

Some participants also provided open-ended feedback, though not all responded. Insights from both the interviews and the questionnaire participants are summarized by criterion below. Additional qualitative feedback and corresponding refinements are presented in Supplementary Tables 3–5.

- **Ease of use**
  This criterion assessed how easy the proposed harmonization process was to follow. Participants consistently rated Ease of Use and Completeness similarly. The questionnaire results (see Table 5), with a mean score of 4.43 (corresponding to “Agree”), suggest that participants generally found the harmonization process easy to use. The interview responses also showed unanimous agreement on this aspect, with participants highlighting the clarity, straightforwardness, and intuitiveness of the process. Example responses included:
  *” I like this because it’s simpler and it’s more for layperson that you don’t have to have a technical background to understand … So I like how you’ve got*,* it’s more straightforward. Like it’s comprehensive … but it’s also not in super technical language”* [IP-7].
  *” I think it is a clear description of the overall process*,* and helps to provide structure to a harmonization process that can be quite complex.”* [QP-6].
- **Completeness**
  This criterion assessed whether the proposed harmonization process adequately covers all essential steps required. The questionnaire results, with a mean score of 4.43, indicated that participants generally felt the harmonization process covered all necessary steps. Most interview participants agreed with this assessment, emphasizing that all critical steps were included. However, some experts suggested areas for improvement, which are summarized in Supplementary Tables 3, 4, and 5 along with the corresponding refinements. Example responses included:
  *” Well*,* it depends on the specific use case*,* but as a general process*,* this is very good. I think this is almost exactly the same as the workflow that I would use and that my colleagues would use.”* [IP-5].
  *” I think all the important steps are covered.”* [QP-5].
- **Efficiency**
  This criterion assessed whether the order and logical structure of the harmonization steps align effectively with real implementation workflows, contributing to an efficient process. The questionnaire results, with a mean score of 4.71 (closer to “Strongly Agree”), were the highest among the other criteria, indicating that participants found the harmonization process to be highly efficient. Interview participants also generally agreed, noting the logical progression of the steps. Example responses included:
  *” I think it was quite logical. I don’t think there was anything out of place*,* per se. So*,* yeah*,* I think it was good.”* [IP-1].
  *” I really like the rationale. I think it’s totally in line with what I have in mind.”* [IP-3].
- **Generality**
  This criterion assessed whether the proposed harmonization process could be generalized across various cancer data types and sources. The questionnaire results, with a mean score of 4.29, were the lowest among the criteria, suggesting that participants found the harmonization process fairly general and adaptable to different cancer data types and sources. Interview responses also supported this aspect, with participants agreeing that the process could be applied to different data types. Example responses included:
  *” As a general process*,* I think it could apply to any data source*,* the way you’re refining it*,* it will be exactly in line with how we as data engineers work with data and with source data. So yeah*,* as a general process*,* this is great. This is exactly how engineers would approach a problem.”* [IP-5].
  *” I think so. I think it’s relevant for all the data sources because you’re not describing something here that’s say data source specific. So*,* I don’t think it’s limiting in any way like that.”* [IP-6].

### Proposed harmonization process

#### Foundational aspects

**Fig. 2:**
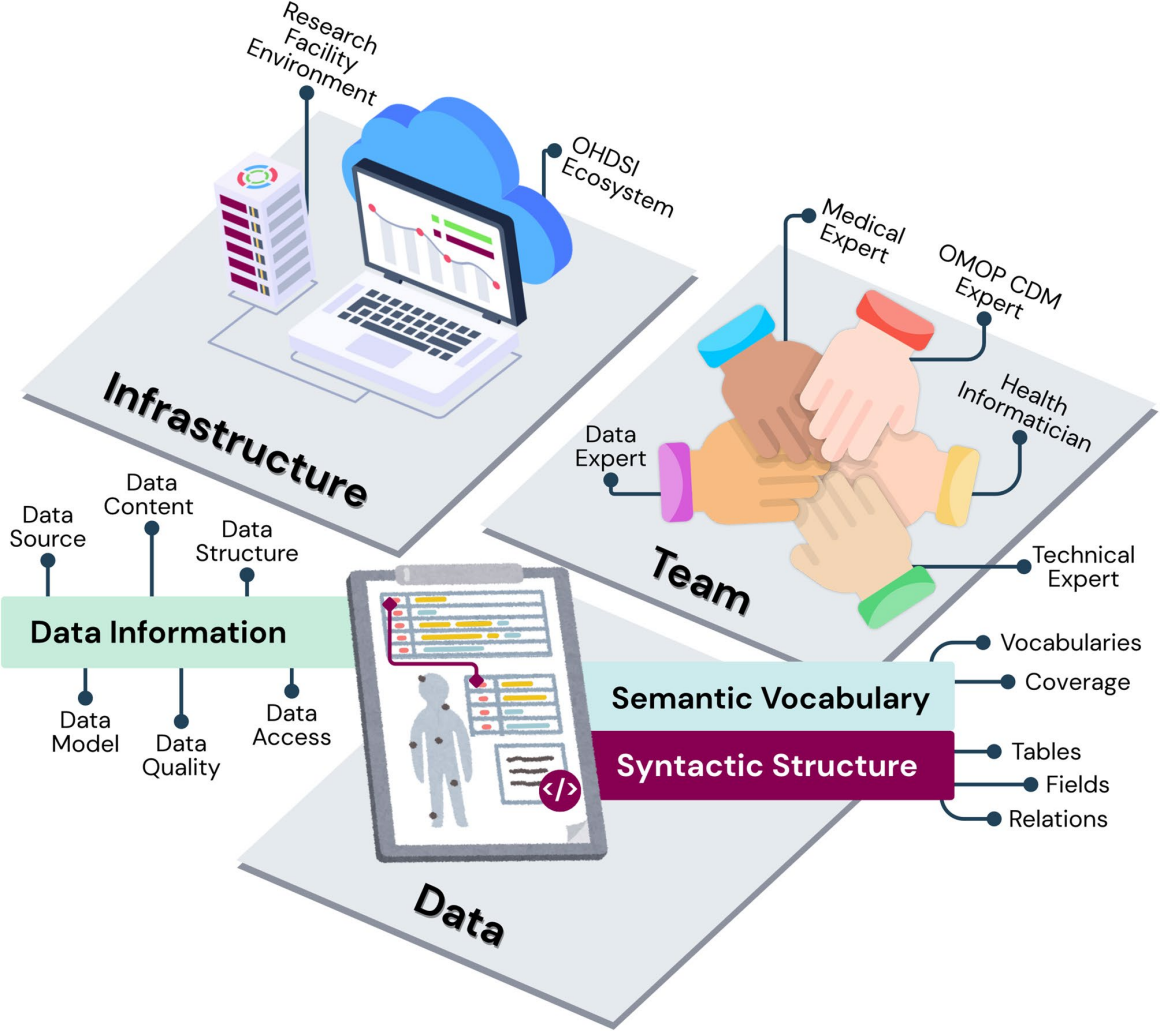
Three interdependent foundational aspects for cancer data harmonization into OMOP CDM. The integration of (1) an interdisciplinary team, (2) a robust infrastructure, and (3) layered data considerations must be considered carefully throughout the workflow, as each element plays a critical role in shaping both the implementation and ongoing refinement of the harmonization strategy.

Harmonizing cancer data into the OMOP CDM is a complex and iterative process built on three interdependent aspects: (1) an interdisciplinary team, (2) a robust infrastructure, and (3) layered data considerations. These elements must be considered throughout the workflow and can significantly influence implementation depending on the research context, resources, objectives, and data governance policies. The prioritization and sequencing of these aspects may vary by use case and their interdependencies mean that focus and order are dynamic and can evolve over time. Figure 2 illustrates how these three components collectively influence the harmonization process.

#### Team

Cancer data harmonization requires a multidisciplinary team with clearly defined roles. Key contributors may include, but is not limited to, Data Experts, Medical Experts, Technical Experts, OMOP CDM Experts, and Health Informaticians.

Health Informaticians work at the intersection of clinical practice, data, and information technology, aligning standards (e.g., FHIR, OMOP CDM), optimizing data models and workflows, and ensuring semantic interoperabilty^52^. Unlike Data Experts (data management and analysis), Medical Experts (clinical domain knowledge), Technical Experts (infrastructure and pipelines), and OMOP CDM Experts (CDM structure and vocabulary conventions), Health Informaticians integrate these perspectives bridging clinical requirements with technical implementation, while addressing governance and interoperability^52^.

These roles are complementary and my overlap within individuals. Team composition should be adapted to project needs, but clear role delineation improves efficiency and ensures both technical rigor and clinical relevance. Active engagement with OHDSI community is strongly recommended to stay aligned with OMOP CDM updates and methodological developments, as well as to leverage collective expertise when addressing harmonization challenges.

#### Infrastructure

Robust infrastructure is essential for effective harmonization. The OHDSI community provides a suite of tools and algorithms (Table 2) that should be leveraged where permitted. However, additional institution-specific infrastructure may be required depending on the project context. Infrastructure planning should include requirement analysis, governance compliance, and personnel expertise. This typically involves establishing database environments, servers, security protocols, Extract-Transform-Load (ETL) and data quality assessment workflows. Supporting technologies such as Docker, SQL, and R/Python environments may also be necessary to enable transformation and quality assurance processes.

#### Data

Data is the core of the harmonization process. Its characteristics, such as source, content, structure, model adherence, quality, and access constraints directly influence implementation. Two primary layers must be addressed: (1) the data information layer and (2) the standardized layer (semantic vocabulary and syntactic structure).

- **Base Layer: Data Information**
  This foundational layer involves understanding source data characteristics to guide harmonization decisions. The data information layer comprises the following:
  **Data Source Types**: Sources may include EHRs, cancer registries, biobanks, claims databases, and PROs, each with unique characteristics and challenges.
  **Data Content**: The content of the data must be assessed to determine whether it requires aggregation or abstraction. Data can be categorized as either:
    **Atomic data**: directly mappable elements (e.g., tumor size, lab values), requiring no abstraction. Some domains (e.g., genomics) may require specialized tools like KOIOS to map it to OMOP CDM with OMOP Genomic vocabularies.
    **Abstracted data**: require aggregation or development of specific algorithms to derive meaningful insights, such as treatment regimens requiring aggregation via tools like ARTEMIS and OncoRegimenFinder.
  **Data Structure**: The data structure can be categorized as structured (e.g., relational databases, cancer registries), semi-structured (e.g., pathology reports), or unstructured (e.g., free-text clinical notes); the latter two require substantial preprocessing before harmonization.
  **Data Model Adherence**: Alignment to a specific data model, such as HL7 FHIR or openEHR, affect transformation complexity and may require dedicated conversion tools.
  **Data Quality**: Issues such as duplication, discrepancies, missing values, outliers, ambiguity in abstraction, noise, and problems with data provenance, metadata, reliability, data linkage, granularity differences, and negation values, especially those frequently encountered in PROs must be addressed before harmonization. **V**erifying of variables essential to the research question is critical. Addressing data quality issues requires establishing agreed-upon conventions within the team.
  **Data Access**: Governance and privacy restrictions may limit access may, necessitating synthetic datasets that closely mimic the characteristics of the source data for development and testing, with careful attention to fidelity.
  A comprehensive understanding of this layer determines preprocessing requirements and guides subsequent harmonization steps.
- **Standardized Layer: Semantic Vocabulary and Syntactic Structure**
  Following assessing source data, harmonization addressed standardization within the OMOP CDM, either sequentially or in parallel.
**Semantic Vocabulary**
    This layer maps source concepts to OMOP standardized vocabularies. Non-standard or local country-specific terminologies (e.g., billing codes) may require translation or local code dictionaries. OMOP conventions define concept domains and table placement, which must be followed. For oncology, cancer diagnoses are typically coded as pre-coordinated concepts that combine histology and topology using International Classification of Diseases for Oncology, 3rd edition (ICD-O-3) and Systematized Nomenclature of Medicine Clinical Terms (SNOMED CT). Understanding these conventions is crucial to ensure accurate and consistent semantic mapping.
**Syntactic Structure**
    This layer aligns source data structure (tables, fields, relationships) within OMOP’s person-centric model. Structural mapping follows domain categorization, ensuring correct table integration.
    For cancer data, the Episode and Episode_event tables are critical for representing longitudinal trajectories, such as treatment regimens and disease progression, and must be appropriately linked to relevant OMOP tables (e.g., Condition_occurrence, Drug_exposure, or Procedure_occurrence).

#### Harmonization process principles

Two key principles guide the harmonization process: iteration and comprehensive documentation.

- **Iterative Nature**
  Harmonization is inherently iterative, requires cyclical refinement based on mapping validation, evaluation results, and optimization. Continuous feedback loops ensure semantic (meaning of the data) and syntactic (format and structure) consistency when transforming cancer data into the OMOP CDM. This iterative approach supports accuracy, clinical relevance, ongoing updates, and improved data capture over time.
- **Documentation**
  Comprehensive documentation ensures transparency and reproducibility. All steps, assumptions, mapping decisions, agreed conventions, transformation rules, technical specifications, and version changes should be recorded. Issues and their resolutions must also be logged. This documentation facilitates future optimization, traceability, and sustained harmonization efforts.

### Harmonization process steps

**Fig. 3:**
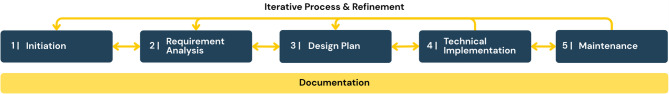
Proposed cancer harmonization process steps into OMOP CDM with iterative and documentation principles. The workflow is structured around a series of interconnected steps that guide the transformation of raw oncology data into a standardized format suitable for large-scale analysis. Each step incorporates feedback loops to support continuous refinement and adaptation based on evolving research needs, data quality assessments, and stakeholder input.

The proposed harmonization process comprises five steps: Initiation, Requirement Analysis, Design Plan, Technical Implementation, and Maintenance. Each step embeds iterative refinement and documentation. Figure 3 illustrates the proposed harmonization process steps, emphasizing the iterative nature and the importance of comprehensive documentation.

**Fig. 4:**
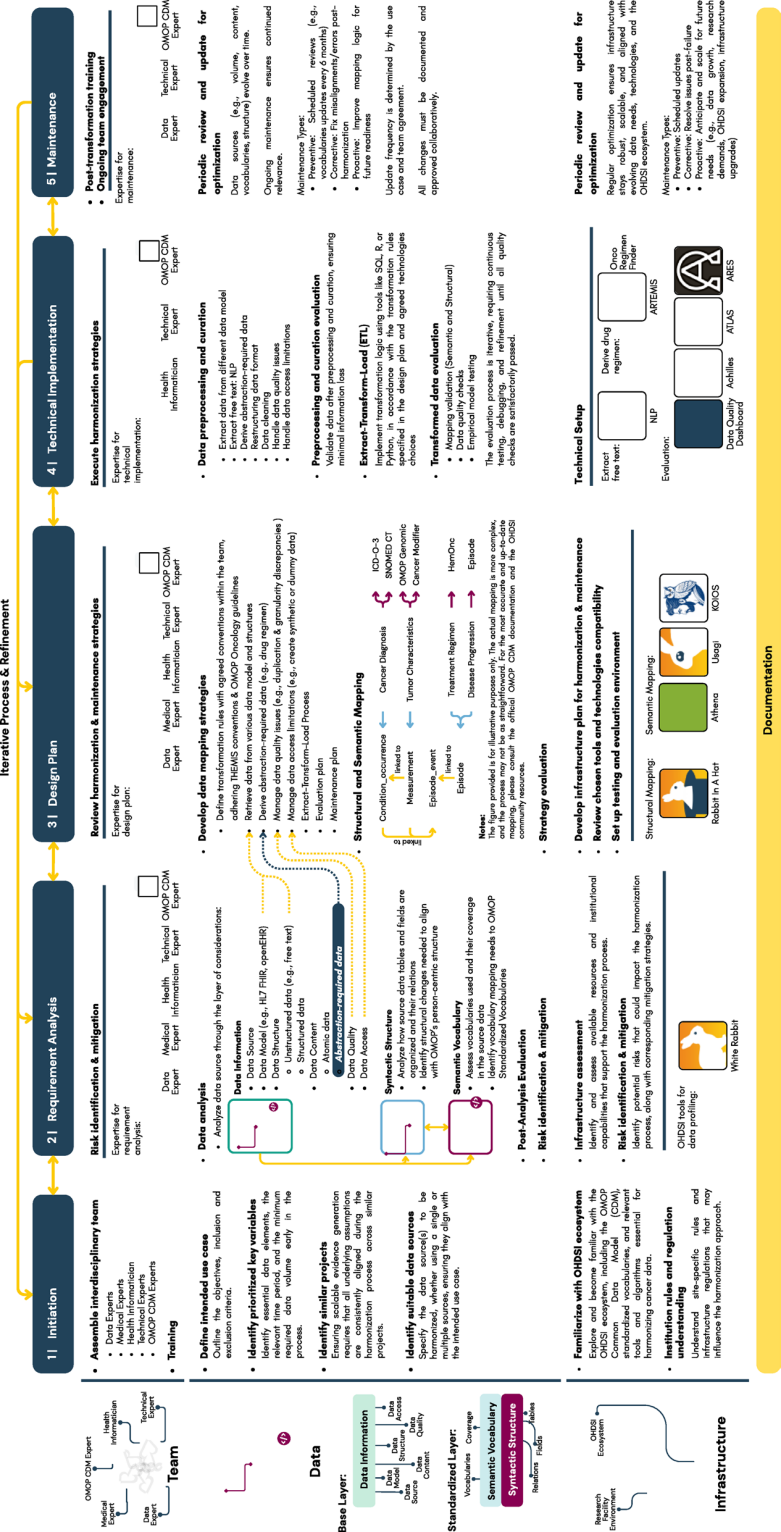
Overview of the cancer data harmonization process into OMOP CDM. The process is structured around three foundational aspects − (1) an interdisciplinary team, (2) a robust infrastructure, and (3) layered data considerations, presented on the rows, - and five key steps - presented in the columns. The workflow is further guided by core principles of iteration and documentation, ensuring adaptability, transparency, and reproducibility.

Additionally, Supplementary Table 6 details the integration of the three foundational aspects across steps with cancer-specific adaptations, while Fig. 4 presents an overall framework combining the foundational aspects, the principles, and five step workflow.

## Discussion

The literature review revealed a notable gap in cancer data harmonization research, with only nine academic papers specifically addressing transformation into the OMOP CDM, consistent with prior findings^7^. To address this limitation, gray literature was included, resulting in 20 total sources from academic databases, the OHDSI website, and Google Scholar.

Most studies relied on cancer registries and EHRs, while PROs and biobank data were less frequently utilized. Consistent with prior research^7^, EHRs were the most common source. However, cancer registries were more frequently represented than reported by Wang et al.^7^, likely due to the inclusion of gray literature; notably, six out of ten registries-based studies originated from gray literature, indicating ongoing efforts in this area are not always captured in peer-reviewed publications.

Significant gaps remain in harmonizing of PROs and genomic data. PROs are challenging due to incomplete or optional responses^53^, and their integration into OMOP CDM remains limited. Genomic data harmonization is also still evolving, despite the development of OMOP Genomic Vocabularies (2020)^54^ and the KOIOS mapping tool (2023)^47^. However, the limited adoption of KOIOS reflects the early stage of integration for genomic data in cancer research.

Regarding cancer type, most publications did not specify a particular type and instead reported “general cancer” data. This contrasts with prior findings^7^ which reported fewer unspecified types. Furthermore, Wang et al.^7^ found that most studies utilized a single dataset, this study observed a more balanced use of single and multiple datasets.

Regarding geographical representation, 70% of the included articles utilized European clinical data, with fewer from the Americas and Asia. This likely reflects publication patterns and coordinated regional initiatives supporting OMOP CDM adoption, such as European Health Data & Evidence Network (EHDEN)^8^ and several cancer-focus programs (e.g., PIONEER^9^, BlueBerry^10^, FLORENCE^11^, IDEA4RC^12^, ONCOVALUE^13^, VALO^14^, rather than definitive evidence of greater uptake. In contrast, Wang et al.^7^ reported higher representation from the Americas and Asia; this variation likely stems from this study’s inclusion of gray literature focused specifically on harmonization, whereas Wang et al.^7^ considered a wider variety of research contexts. Regional differences in governance, standards, and health system organization may also influence harmonization design and implementation, limiting generalizability and highlighting the need for broader international representation.

Additionally, the study identified limited use of recently developed OHDSI regimen derivation tools, such as OncoRegimenFinder (last updated in 2021)^46^ and ARTEMIS (2023)^47^; likely due to their recent release and early stage of cancer harmonization efforts. However, tools like Athena, ATLAS, DQD, Usagi, White Rabbit, Achilles, and Rabbit In A Hat were frequently used. Interestingly, no studies have explicitly analyzed the frequency of OHDSI tool usage in harmonization, highlighting a gap in the literature.

A major gap concerns the harmonization of multiomics, imaging, and emerging data models (e.g., HL7 FHIR, openEHR) within cancer context. Cancer data are inherently multimodal, integrating multiomics layers (e.g., genomics, transcriptomics, proteomics, epigenomics), imaging, radiomics, and radiogenomics, which introduce substantial integration challenges^55–57^. These complexities are only briefly addressed in current harmonization pipelines. Expert feedback reinforced this finding and underscores the need for future research to address the harmonization of multiomics and imaging data, as well as the integration of emerging data models.

Although the OHDSI community is actively developing extensions for multiomics^58^, imaging^54,59,60–65^,PROs^53,66^, and FHIR to OMOP transformation tools (e.g., eXtensible Stylesheet Language Transformations (XSLT) processors to transform HL7 FHIR Bundles into OMOP CDM schema^67^, the FHIR subscription feature for real-time data transformation^68^, and OMOP-on-FHIR tools^69^, these efforts remain under active development. Similarly, integration efforts between openEHR and OMOP CMD (e.g., OMOP Conversion Language (OMOCL) and the Eos tool^70^ are evolving. The proposed process addresses this dynamic landscape through its Maintenance step, emphasizing iterative adaptation to technological advances.

Finally, while interoperability frameworks such as FHIR and openEHR were considered, oncology-specific models such as minimal Common Oncology Data Elements (mCODE)^71^ were not systematically examined. Recent studies have demonstrated the pragmatic integration of mCODE and OMOP CDM within unified institutional data architectures^72^, this represents an important direction for future research.

A consistent similarity across all reviewed studies was the presence of an intended use case for harmonization, although this was not always explicitly stated. In many cases, this intent was inferred from inclusion criteria, data sources, or the stated purpose of the work. Thus, the categories identified reflect each study’s dominant framing rather than mutually exclusive purposes. Additionally, every study described the execution of an Extract-Transform-Load (ETL) process to map cancer data into the OMOP CDM, forming the core of the harmonization effort.

However, differences emerged in how explicitly harmonization steps were reported. Only half of the articles discussed setting up and familiarizing themselves with the OHDSI ecosystem, and approximately 45% mentioned forming an interdisciplinary team. In contrast, data source analysis was addressed in 90% of the articles, data preprocessing in 65%, and quality control in 90%. Ongoing maintenance practices, such as vocabulary updates and tool adaption, were reported in only four studies.

These findings underscore a lack of standardized methodology and highlight areas where harmonization practices diverge. Nevertheless, it is important to note that the lack of explicit reporting does not imply that certain steps were not conducted. For instance, while the formation of an interdisciplinary team was infrequently documented, expert feedback confirmed it as routine and essential in practice, highlighting the inherently multidisciplinary nature of cancer data harmonization.

Several key challenges were identified and categorized into three themes: source data quality and complexity, mapping issues, and maintenance issues. Among these, data mapping emerged as a primary challenge, frequently leading to semantic loss due to incomplete or incorrect mappings, often linked to limited vocabulary granularity in OMOP, a problem also highlighted in previous studies^7^. Maintenance-related challenges were also highlighted as critical but often overlooked area, underscoring the need for continuous optimization to accommodate evolving cancer data requirements and technologies.

Five major themes emerged that form the foundation of the proposed harmonization process: initiation, requirement analysis, design planning, technical implementation, and maintenance. These steps reflect recurring patterns and challenges identified in the literature review.

**Fig. 5:**
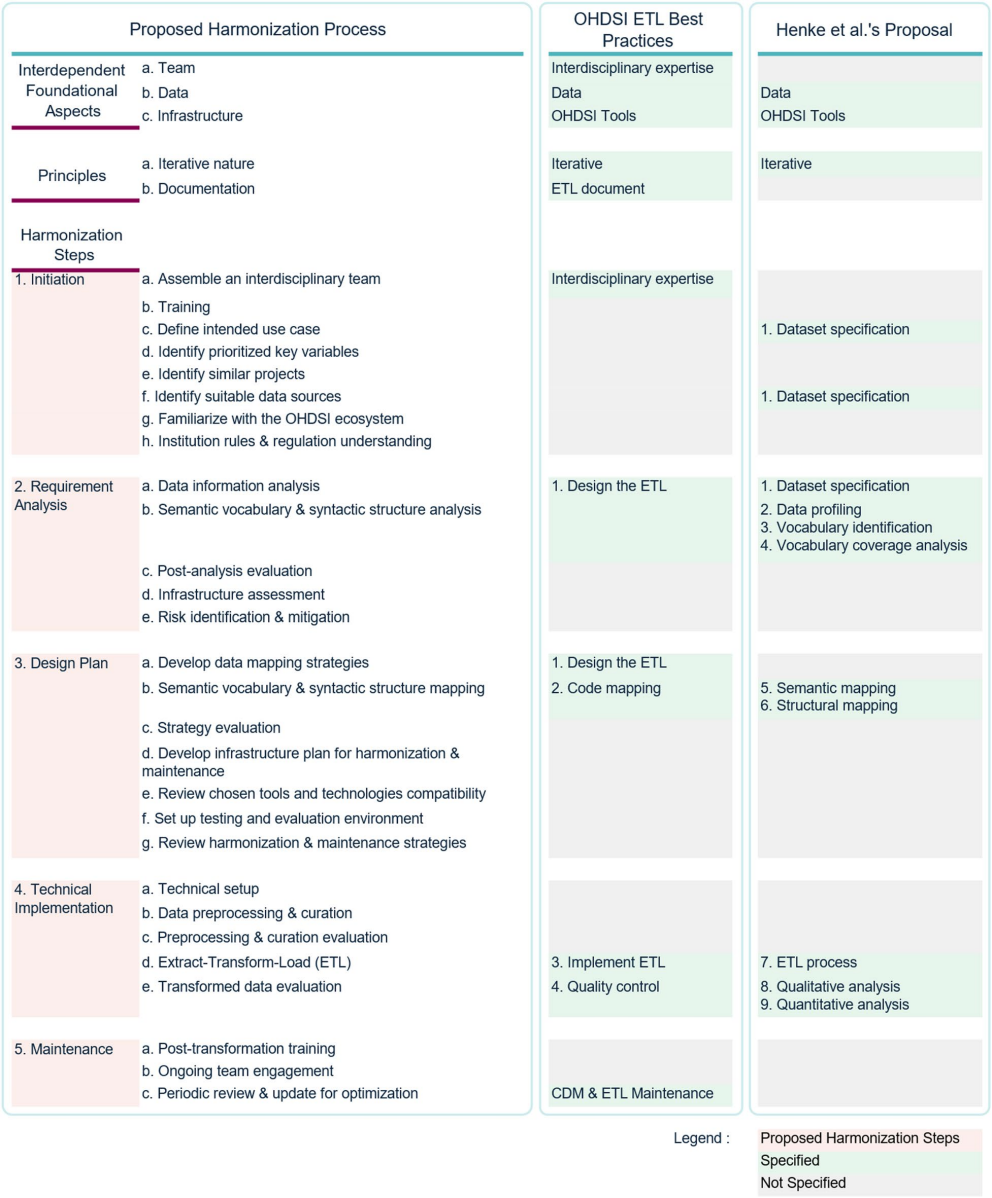
Overview of the proposed cancer data harmonization process into OMOP CDM in comparison with existing frameworks. Numbered statements under OHDSI ETL Best Practices^21^ and Henke et al.‘s Proposal^22^ indicate the explicit sequence of steps within each methodology, while unnumbered statements represent information that is implicitly derived or not explicitly stated in the original methodological steps.

Comparison with two existing methodologies (Fig. 5): OHDSI’s ETL best practices^21^ and Henke et al.’s nine-step framework^22^ showed overall structural alignment but revealed two key divergences: explicit “Initiation” and “Maintenance” steps, which are not explicitly addressed in either of the frameworks. This study also confirmed that many reviewed studies described ad hoc approaches shaped by site-specific constraints, reinforcing the importance of these additional phases for addressing the complexity and ensuring long-term sustainability of cancer data harmonization.

The proposed process integrates insights from the literature review and existing frameworks from OHDSI^21^ and Henke et al.^22^, further refined through feedback from academic and domain experts to enhance practical applicability and methodological soundness.

The final design of the proposed process introduces three interdependent foundational aspects: Data, Team, and Infrastructure. While the OHDSI framework^21^ implicitly addresses data and team aspects, and Henke et al.^22^ address the data aspects, neither framework sufficiently considers institutional and research infrastructure, which plays a critical role in the real-world implementation of data harmonization. This study emphasizes infrastructure as an integral aspect at every step, encompassing both the OHDSI ecosystem and broader institutional environment required for real-world implementation.

Within the data aspects, two layers are distinguished: (1) data information layer and (2) semantic vocabulary and syntactic structure layer. While the semantic vocabulary and syntactic structure layer is explicitly addressed in both the OHDSI^73^ and Henke et al.’s frameworks^22^, given that OMOP CDM is designed to standardize both the structure and semantics of observational data^73^ , they do not explicitly examined the data information layer. This study highlights its importance as understanding source characteristics is essential for accurate and context aware.

**Fig. 6:**
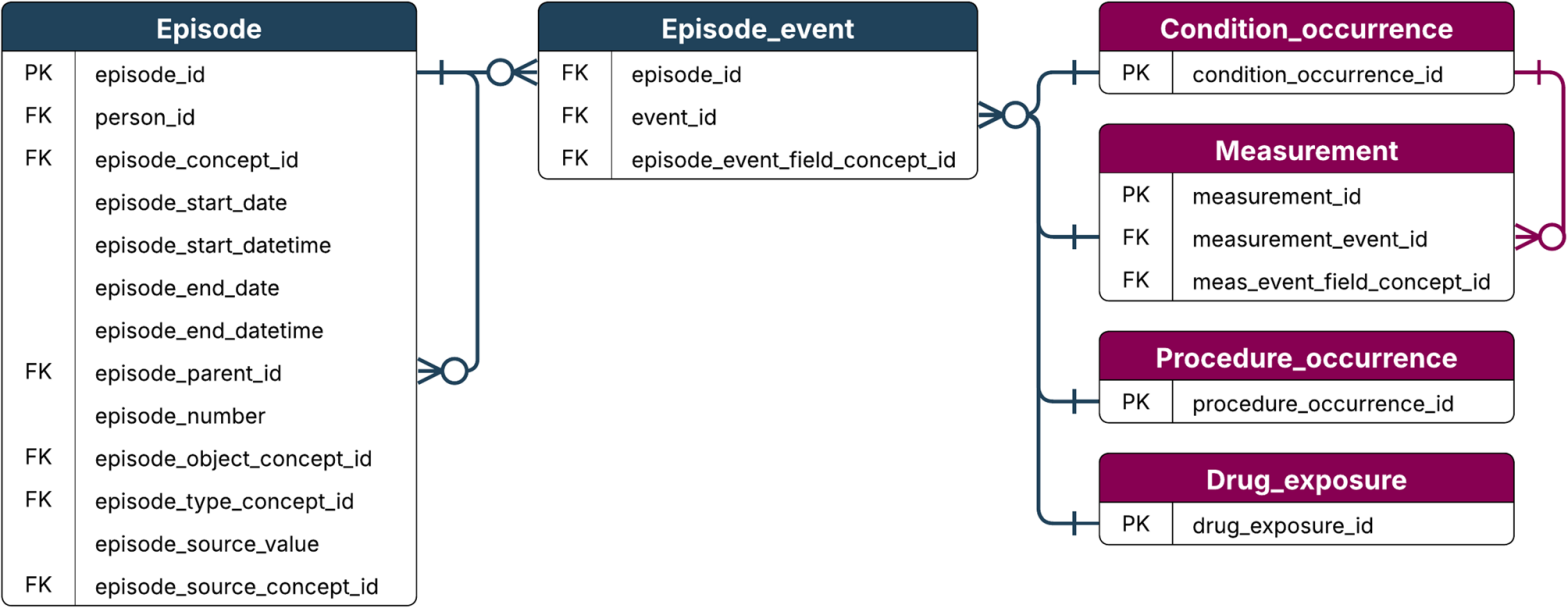
Episode and Episode_event tables attributes and relation to other tables, adapted from^74,75,76^ with modifications. To accurately capture the longitudinal trajectory of cancer episodes, oncology-specific elements, such as treatment regimens, staging, and disease progression, require the use of specialized tools and algorithms. These components must be carefully mapped into the Episode table and appropriately linked to the Episode_event table, which is specifically designed to represent the temporal and clinical progression of cancer care. PK = Primary key; FK = Foreign key.

Additionally, while both frameworks acknowledge the iterative nature of harmonization, they do not specifically emphasize on comprehensive documentation. In contrast, this study underscores documentation as a central principle to ensure transparency, reproducibility, traceability, and long-term sustainability. This study specifically focuses on harmonizing cancer data. While the proposed process aligns with existing frameworks in several ways, it also introduces cancer-specific considerations within data aspects, particularly the need to address the complexities of cancer data, such as treatment regimens, staging, and disease progression, which may require data abstraction to derive this information. These elements are not typically found in other medical conditions and require specialized tools and algorithms for accurately mapping into the Episode table and linking it into the Episode_event table specifically designed to capture cancer episode trajectories (Fig. 6). This step reflects the cancer-specific nature of the harmonization process.

Another key difference of the proposed process is that it considers the practicality. Beyond being grounded in a literature review of identified patterns and challenges, it was refined through feedback from domain experts with hands-on experience in cancer harmonization. This ensures real-world relevance. For instance, the introduction of “Data Preprocessing & Curation” activity in “Technical Implementation” step (Fig. 4) addresses recurrent challenges such as information loss, manual curation of unstructured data^27–31,35,38,39,42^, and handling non-standard vocabularies, molecular data, and PROs^24–26,28–33,35,39,40^. The heterogeneity and complexity of cancer data further justify this dedicated activity.

Furthermore, expert feedback led to the introduction of practical elements not commonly reported in prior studies. These include the need for team training, familiarization with the OHDSI ecosystem, identification of similar projects to ensure scalable evidence generation, and early consideration of institutional regulations. Together, these considerations strengthen the “Initiation” step, highlighting its importance for successful implementation (Fig. 4).

In addition, the “Risk Identification & Mitigation” activity within the “Requirement Analysis” step has been further emphasized in response to academic expert feedback. Although not explicitly addressed in existing frameworks^21,22^, this activity is essential given the complexity of cancer data harmonization. It is therefore treated as a distinct activity within the proposed process. According to ISO 31000:2018^77^, the risk management process includes establishing the scope and context, conducting risk assessment (comprising identification, analysis, and evaluation), implementing risk treatment, recording and reporting, monitoring and review, and engaging in communication and consultation. However, the proposed process does not prescribe a specific methodology, allowing flexibility based on the intended use case, infrastructure, data characteristics, team composition, and other contextual factors.

Maintenance, although discussed in the OHDSI books^21^, is not explicitly incorporated into OHDSI’s ETL best practices (Fig. 5). Given continuous updates to OMOP CDM, standardized vocabularies, and related tools (e.g., Episode and Episode_event tables, Cancer Modifier and OMOP Genomic vocabularies, KOIOS, OncoRegimenFinder, ARTEMIS), ongoing monitoring and adaptation are critical. The literature and expert feedback both emphasized that maintenance extends beyond operational continuity to include preventive, corrective, and optimization efforts^78,79^. This dedicated phase ensures that harmonized cancer data remain accurate, standardized, and sustainable in a rapidly evolving research and technological landscape.

One notable strength of the study is its methodological triangulation. By integrating academic and gray literature, it provides a comprehensive overview of cancer data harmonization and captures ongoing efforts not yet reflected in peer-reviewed publications. The identified patterns and challenges directly informed the proposed harmonization process, ensuring that it addresses recurring gaps in existing methodologies. Another strength lies in the three iterative refinement rounds involving academic and domain experts. Their feedback enhanced both methodological rigor and practical applicability. Notably, experts input led to the inclusion of activities underrepresented in prior frameworks, such as team training, familiarization with OHDSI ecosystem, identification of related projects, and early consideration of institutional regulations, reinforcing the importance of a dedicated “Initiation” step.

However, several limitations should be acknowledged. Selection bias may have occurred during literature inclusion, particularly given the author’s initial limited familiarity with cancer data and OMOP CDM. In the evaluation phase, only eight experts completed the questionnaire, with one excluded due to “Unable to Answer” responses, reducing the quantitative sample to seven and limiting statistical robustness. Although responses were generally positive, the small sample constrains generalizability.

The interview phase also did not fully reach data saturation, likely due to the multifaceted nature of cancer data and the diversity of participant expertise. Moreover, most participants had technical backgrounds, with fewer medical experts represented, resulting in feedback that was more technical oriented. Future studies should include a larger and more diverse expert pool.

Another limitation is the limited consideration of the national origin of source data with which the experts had worked. Although OMOP has since evolved into a global framework under OHDSI initiative^73^, it was initially developed to support clinical data from U.S. settings^73,80^. Differences in national coding systems, vocabulary granularity, and pathology reporting standards may introduce additional semantic harmonization challenges, particularly for non-U.S. contexts. These cross-national interoperability issues warrant more systematic investigation. Recent efforts to harmonize U.S. and European cancer pathology reporting standards using SNOMED CT^81^ further illustrate the complexity of achieving cross-national semantic interoperability in oncology data. Despite these limitations, expert feedback substantially strengthened the framework. While the latest iteration was not revalidated by domain experts, it underwent academic review to ensure methodological and theoretical soundness.

In this study, the harmonization process was evaluated using four criteria: ease of use, completeness, efficiency, and generality. While informative, these criteria are not exhaustive. The Design Science Research Methodology (DSRM) applied in this research does not provide standardized guidance for artifact evaluation, leaving room for interpretive variability. For example, “efficiency” may be understood as problem-solving effectiveness or as structural/logical design efficiency. This ambiguity limits the precision with which the evaluation can be conducted. More detailed guidelines and a broader set of evaluation criteria would help ensure consistency and accuracy in future assessments. Generality was assessed in terms of applicability across diverse cancer data types and heterogeneous data sources, but not across distinct intended use cases (e.g., clinical and translational research versus population health studies and analytics). Future evaluations should extend the concept of generality to include adaptability across research purposes.

The evaluation targeted domain experts with direct experience in cancer data harmonization into OMOP CDM. While appropriate, this focus may have excluded other relevant stakeholders, including those involved in ongoing but unpublished initiatives or individuals planning for future OMOP implementation. Including such groups could provide additional insights into clarity, usability, and broader applicability.

The study’s findings have important implications for research and practice. The proposed harmonization process offers a structured yet flexible approach for integrating heterogeneous cancer data (e.g., EHRs, registries, claims) into OMOP CDM. By emphasizing data preprocessing and curation, and acknowledging specialized oncology tools (e.g., OncoRegimenFinder, ARTEMIS, KOIOS), it directly addresses known challenges in cancer data transformation. Its adaptability support both novice and experienced users.

The need for such framework stems from the steep learning curve associated with OMOP CDM^82^, particularly in oncology. Existing guidance across OHDSI and academic sources is often fragmented and not tailored specifically to cancer data. Additionally, the OHDSI book guidelines were first published nearly six years ago^83^, with the latest update to the online version occurring in 2021^84^. As a result, it has not fully incorporated recent oncology-specific developments. This proposed harmonization process addresses that gap by offering a comprehensive, stepwise, and adaptable framework capable of evolving alongside advances in oncology research and interoperable health data infrastructures.

### Methods

#### Research approach

This study follows the Design Science Research Methodology (DSRM)^85^ to create an artifact, a generic harmonization process for cancer data into the OMOP CDM, addressing the lack of a standardized approach for cancer-specific data harmonization. DSRM follows an iterative development and evaluation process, ensuring the artifact’s robustness and effectiveness^85^. Supplementary Fig. 2 shows the DSRM process and its iterations that are applied in this study. The study was processed through the six phases of the DSRM^85^:

1. **Defining the Problem and Motivation**:
  The existing frameworks for harmonization data into OMOP^21,22^ lack specificity for oncology data and do not address challenges related to unstructured text extraction, highlighting the need for a tailored harmonization process for cancer data.
2. **Describing the Objectives of the Solution**:
  The main objective is to design a generic harmonization process for cancer data into the OMOP CDM. The proposed solution aims to be adaptable, comprehensive, and applicable to diverse cancer datasets, supporting the effective secondary use of oncology data for research purposes.
3. **Designing and Developing the Artifact**:
  The artifact, a generic harmonization process, was conceptualized and developed through thematic and content analysis of the literature and built upon existing harmonization frameworks.
  A literature review was conducted from 2nd February 2025 to 6th March 2025, drawing from both academic and gray literature to analyze existing cancer data harmonization practices. Given the variability in cancer names, the search strategy accounted for all possible cancer names^86,87^. Supplementary Table 7 detailed the search strategies. A multi-stage screening process was applied for academic literature, including titles, abstracts, and full-text reviews, to ensure the inclusion of relevant studies discussing the cancer data harmonization process into OMOP CDM.
  The search was supplemented by a manual search of the OHDSI website^88,89^ to include gray literature from ongoing projects and symposiums to capture emerging and practical insights that might not yet be published in peer-reviewed databases. Given that abstracts are often unavailable in gray literature, screening was conducted at the title and full-text levels. Additionally, a Google Scholar search was conducted particularly to complement the manual search conducted on the OHDSI website for gray literature, helping to minimize the risk of missing relevant articles. The inclusion of both academic and gray literature ensured a balanced view of theoretical foundations with real-world applications.
  The inclusion and exclusion criteria (Supplementary Table 8) ensured that only studies explicitly discussing OMOP, cancer, and harmonization processes were considered. Zotero software^90^ was used to manage references and remove duplicate articles. The review was reported using the Preferred Reporting Items for Systematic Review and Meta-Analyses (PRISMA) guidelines^91^ to ensure transparency and reproducibility.
  The collected data underwent two complementary analytical approaches: inductive thematic analysis and deductive content analysis, ensuring data-analysis triangulation for robust insights and a comprehensive understanding of cancer data harmonization methodologies. Inductive thematic analysis was valuable for identify recurring themes that the literature may not explicitly state^92–94^, such as harmonization steps, OHDSI tools, and key challenges, using NVivo software^95^. To complement and validate the thematic findings, deductive content analysis^94,96,97^ was applied to quantify the frequency of identified themes, harmonization steps, and tools, offering a structured overview of existing practices and highlighting areas of alignment and divergence.
4. **Demonstrating the Artifact**:
  Due to resource constraints, the artifact could not be empirically tested. To further illustrate its practical relevance and feasibility, a hypothetical scenario was developed and applied. This scenario was designed based on insights gathered from the literature review. While specific implementations of HL7 FHIR and openEHR were not explicitly discussed in the reviewed literature, their growing significance in the healthcare interoperability landscape makes them important to consider. Therefore, the scenario was intentionally crafted to incorporate the potential utilization of HL7 FHIR and openEHR, reflecting anticipated future directions in health data standardization and integration. By including these data models, the scenario extends beyond the current literature to explore how the artifact could remain adaptable and relevant as data standards evolve. A full overview of the detailed demonstration can be seen in Supplementary Data 1.
5. **Evaluating the Artifact**:
  The artifact evaluation relied on expert feedback rather than formal testing. SB author assessed the artifact’s theoretical and methodological soundness, while domain experts with experience in harmonizing cancer data into OMOP CDM evaluated its practical applicability. The illustrative scenario method^98^ from the demonstration phase was utilized to assess the artifact’s suitability. Additionally, domain expert evaluation^98^ was conducted using a mixed-methods approach, combining semi-structured interviews (Supplementary Data 2) for qualitative insights and a Likert-scale questionnaire (ranging from Strongly Disagree to Strongly Agree) (Supplementary Fig. 3) for quantitative assessment. This methodological triangulation enhanced the credibility of the evaluations^99,100^.

#### Sampling strategy and data analysis

A purposive sampling strategy^101,102^ was used to select domain experts based on their expertise and experience in harmonizing cancer data into OMOP CDM. Inclusion criteria include direct involvement in OMOP CDM cancer data harmonization, demonstrated through publications, posters, or participation in relevant projects. Exclusion criteria include individuals without direct involvement or those unable to participate due to time constraints or other commitments. The target sample size for interviews is a minimum of nine participants, based on empirical findings from literature reviews^103,104^, meaning no new evaluation elements emerge.

For qualitative data analysis and management, Microsoft Teams^105^ was used to conduct the interview and transcribe interviews verbatim with manual review to ensure their correctness, and NVivo software^95^ was used to allow effective coding, rich text analysis, and compatibility with thematic analysis^106,107^. Thematic analysis was employed to identify recurring themes from the expert feedback^92,93^.

#### Evaluation criteria, process, and iterative refinement

The evaluation focused on specific method artifact evaluation elements suggested by March & Smith^108^,including operationality, efficiency, generality, ease of use, completeness, and consistency. Nevertheless, given the conceptual nature of the proposed harmonization process, which has not yet been empirically implemented, the study only assessed criteria that experts can reasonably evaluate based on their experience at this stage. Therefore, the artifact was evaluated on ease of use, completeness, efficiency, and generality. Criteria such as operationality and consistency, which typically require empirical implementation for accurate assessment, were not evaluated at this stage.

Each round of expert feedback informed iterative refinements of the artifact, following the DSRM iterative process to ensure a robust final version, as outlined below:

1. **Preliminary Evaluation**: An academic expert conducted an initial review of the proposed harmonization process, focusing on its overall structure and theoretical foundation. The feedback informed the first refinement of the artifact.
2. **Domain Experts’ Evaluation**: Domain experts provided practical feedback through online interviews conducted via Microsoft Teams^105^ and questionnaires distributed via Microsoft Forms^109^.
3. **Final Evaluation**: A final comprehensive review was conducted by SB author (the academic expert), focusing on theoretical soundness and methodological rigor. This feedback informed the final refinement of the harmonization process.

## Communicating the outcomes

The findings were documented to share insights with the broader research community.

## Ethical considerations

The study was carried out in Sweden. According to the Swedish Ethical Review Act (SFS 2003:460)^98^ and guidance from the Swedish Ethical Review Authority^99^, the type of research presented in this manuscript does not require formal ethical approval, as it does not involve sensitive personal data as defined by the EU General Data Protection Regulation (EU 2016/679)^100^. Nonetheless, we emphasize that all ethical standards were strictly followed in accordance with relevant legislation, the Declaration of Helsinki^101^.

Participants, i.e., domain experts, were fully informed about the study’s purpose, their rights, and data handling procedures. Informed consent was obtained from all human research participants. Participation was voluntary, and withdrawal was permitted at any time. Anonymity was guaranteed, and participants were informed that findings would be publicly disseminated.

## Supplementary Information

Below is the link to the electronic supplementary material.

Supplementary Material 1

## Data Availability

All data generated or analysed during this study are included in this published article [and its supplementary information files].
