# Supplementary material for: Data harmonization processes of cancer data into the observational medical outcomes partnership common data model
Source: Sci Rep. 2026 May 22;16:15993. doi: 10.1038/s41598-026-53570-9 (PMC13197402; doi:10.1038/s41598-026-53570-9)
Supplement: Supplementary file 1 — Supplementary Material 1 [file 41598_2026_53570_MOESM1_ESM.pdf]

Supplementary Information

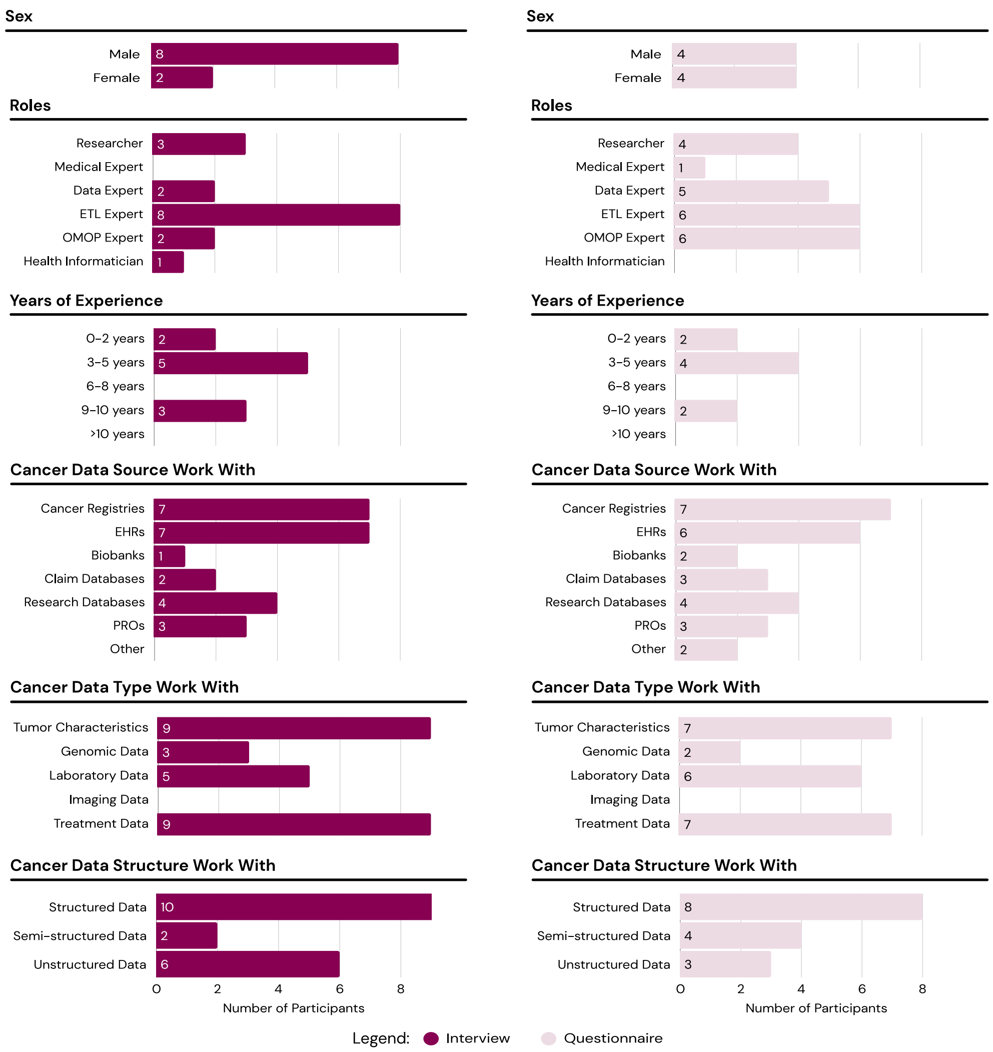

Supplementary Fig. 1 | Domain expert participant characteristics. Since the questionnaire was anonymous, it is unknown which individuals completed it. Given that some experts expressed willingness to participate in both evaluation methods, it is important to note that there may be overlapping characteristics between the interview and questionnaire participants. However, the interview sessions are primarily focused on exploring participants' insights, so it is worth noting that the characteristics of the participants were not as thoroughly examined as they were in the questionnaire.

Supplementary Table 1 | Individual characteristics of domain expert participant in interview method

Participant	Sex	Roles	Range of Experience	Cancer Data Characteristics Work With
IP-1	Male	ETL Expert	3-5 years	• Cancer registries and EHRs<br>• Cancer diagnosis, tumor characteristics, laboratory, & treatment data<br>• Structured and unstructured data
IP-2	Male	ETL Expert	3-5 years	• Cancer registries, EHRs, and biobanks<br>• Cancer diagnosis, tumor characteristics, laboratory, & treatment data<br>• Structured data
IP-3	Male	Researcher, ETL Expert	3-5 years	• EHRs and research databases<br>• Cancer diagnosis, tumor characteristics, & treatment data<br>• Structured data
IP-4	Male	Researcher, ETL Expert	3-5 years	• EHRs, research databases, and PROs<br>• Cancer diagnosis, tumor characteristics, & treatment data<br>• Structured and unstructured data
IP-5	Male	ETL Expert	0-2 years	• Research databases<br>• Genomic data<br>• Structured data
IP-6	Male	ETL Expert, OMOP Expert	9-10 years	• Cancer registries, EHRs, claim databases, & research databases<br>• Cancer diagnosis, tumor characteristics, genomic data, laboratory, & treatment data<br>• Structured, semi, & unstructured data
IP-7	Female	Data Expert	0-2 years	• Cancer registries<br>• Cancer diagnosis, tumor characteristics, & treatment data<br>• Structured and unstructured data
IP-8	Male	ETL Expert	3-5 years	• Cancer registries, PROs<br>• Cancer diagnosis, tumor characteristics, & treatment data<br>• Structured data
IP-9	Male	Health Informatician	9-10 years	• Cancer registries, EHRs<br>• Cancer diagnosis, tumor characteristics, genomic data, laboratory & treatment data<br>• Structured and unstructured data
IP-10	Female	Researcher, Data Expert, ETL Expert, OMOP Expert	9-10 years	• Cancer registries, EHRs, claim databases, PROs<br>• Cancer diagnosis & tumor characteristics, laboratory, and treatment data<br>• Structured, semi, & unstructured data

Supplementary Table 2 | Individual characteristics of domain expert participant in questionnaire method

Participant	Sex	Age Range	Roles	Range of Experience	Cancer Data Characteristics Work With
QP-1	Male	41-50 years	Researcher, Data Expert, ETL Expert, OMOP Expert	3-5 years	• EHRs & research databases<br>• Genomic, laboratory, and treatment data<br>• Structured data
QP-2	Male	31-40 years	ETL Expert, OMOP Expert	3-5 years	• Cancer registries, EHRs, & biobanks<br>• Cancer diagnosis and tumor characteristics<br>• Structured data
QP-3	Female	>60 years	Data Expert	0-2 years	• Cancer registries & oncological basis dataset<br>• Cancer diagnosis, tumor characteristics & treatment data<br>• Structured and semi-structured data
QP-4	Female	31-40 years	Researcher, Data Expert, ETL Expert, OMOP Expert	3-5 years	• Cancer registries & PROs<br>• Cancer diagnosis, tumor characteristics, laboratory, & treatment data<br>• Structured data
QP-5	Male	31-40 years	ETL Expert	3-5 years	• Cancer registries, EHRs, biobanks, claim databases, research databases<br>• Cancer diagnosis, tumor characteristics, laboratory, & treatment data<br>• Structured, semi, & unstructured data
QP-6	Male	>60 years	ETL Expert, OMOP Expert	9-10 years	• Cancer registries, EHRs, claim databases, & research databases<br>• Cancer diagnosis, tumor characteristics, genomic data, laboratory, & treatment data<br>• Structured, semi, & unstructured data
QP-7	Female	21-30 years	Researcher, Medical Expert, Data Expert, OMOP Expert	0-2 years	• Cancer registries, EHRs, research databases, PROs, & quality assurance data<br>• Cancer diagnosis, tumor characteristics, laboratory, & treatment data<br>• Structured data
QP-8	Female	31-40 years	Researcher, Data Expert, ETL Expert, OMOP Expert	9-10 years	• Cancer registries, EHRs, claim databases, PROs<br>• Cancer diagnosis & tumor characteristics, laboratory, and treatment data<br>• Structured, semi, & unstructured data

Supplementary Table 3 |Academic expert feedback and refinements on the preliminary evaluation

Themes & Subthemes	Feedback	Refinement for the Proposed Process
**General Overview**		
Layer of consideration	*“I would recommend adding explanations on "Layers of Consideration before Data Harmonization.”*	Added an explanation of the "Layers of Consideration" to provide readers with a clearer understanding of the concept.
	“The relationship (if any) between "Layers of Consideration before Data Harmonization" and steps of the harmonization process (steps 2, 3, and 4) should be clarified”	Adjustments were made to the process visualization to ensure a clear distinction between the proposed steps and the three layers of considerations associated with each step. This refinement aimed to eliminate potential overlap and confusion, thereby enhancing the clarity of the presentation for readers.
Technical issue	*“… scanned the QR code, but it appears that the content does not exist, "flip-book problem" error message”*	Removed the QR code from the cover of the flipbook, which was initially included for questionnaire participants to scan and access the proposed process. The QR code led to technical issues, and its removal was necessary to avoid frequent manual updates. Instead, a direct link to the flipbook was provided for participants to access the content seamlessly.

Supplementary Table 4 | Domain experts' feedback and refinements on the second iteration

Themes & Subthemes	Feedback	Refinement for the Proposed Process
General Overview		
Not cancer specific	"*I don't think it was necessarily super oncology dependent.*" [IP-1]<br><br>"*This is a very general approach for all data sets, and you have the specificity for cancer data set because this is what is of interest to you, I assume. But if you remove that, the step three (the cancer data related mapping plan), that is more general (process).*" [IP-4]	Added emphasis on the need for abstraction of the episode of care, a cancer-specific use case, and included specific tools and algorithms for deriving this information, which may not apply to other medical conditions
Iterative refinement	"*It should take into account the need for iterative refinement.*" [IP-3]<br><br>"*One thing that concerns me is that it's direction and there's no obvious way back to the beginning … but these things are by their nature iterative*" [IP-9]	Added information on the iterative refinement process in the proposed harmonization methodology.
Documentation	"*You actually need some kind of agreement in a consortium or in a group. Ideally some kind of guideline document … It's a convention document, basically.*" [IP-6]<br><br>"*If you do mappings, it would also be nice if you can do some kind of keep track of some kind of history … Good documentation is also important.*" [IP-8]	Introduced documentation as an integral part of the harmonization process, including agreements, planning, technical specifications, and tracking history, as updates may occur over time.
**Layers of Consideration**		
Data quality	"*There's a lot of information loss in the medical record related to provenance … It becomes incredibly important to know who contributed and which section of the note … you don't know about the reliability, you don't know why something's missing … it might be worth your while to take a look at it and then to think about how important metadata is.*" [IP-9]<br><br>"*Try to look at the patient's own data to help us determine if we trust the information that's coming forward. And in those cases, we'll then make some sort of determination. We'll come up with an algorithm to decide.*" [IP-10]<br><br>"*There has to be a plan for dealing with redundant and discrepant information … because each data source had a different level of granularity*" [IP-9]<br><br>"*… pieces of information that built upon each other … ended up with inherent ambiguity and inherent disagreements (abstracting cancer information) and you have to have a plan to resolve these things … That's another aspect of ETL that a lot of people don't necessarily consider.*" [IP-9]<br><br>"*There is noise in the medical record … You have to have a method for cleaning those up and recognizing them.*" [IP-9]	Changed the "data completeness" aspect within the Data Information consideration layer to "data quality" to encompass a broader range of quality issues. This adjustment would address challenges related to data provenance, reliability, metadata, redundancy, discrepancies in data sources, ambiguity and disagreement in data abstraction, and noise in data sources, all of which should be carefully considered during the harmonization process.
Syntactic structure &	"*When we talk about these syntactic and semantic mappings … the approach that we*	Refined the layers of consideration to position syntactic structure and semantic

Themes & Subthemes	Feedback	Refinement for the Proposed Process
semantic vocabulary	*sort of use is to actually start with the syntactic part.*" [IP-5]<br><br>"*The rule is always it's whatever you map to, is the concept that decides which table it actually goes to in the end … We do normally do the, we call it just the structural mapping, but we start with that as a starting point. And then actually, often they end up going in parallel.*" [IP-6]<br><br>"*So, in terms of OMOP, the vocabulary really dictates the movement of the data … So I kind of do a little bit of both (syntactic & semantic mapping) ... But, I usually start with vocabulary … I do like a loose vocabulary search, map the tables and fields and then I do like a more strict vocabulary mapping.*" [IP-10]	vocabulary as parallel processes, with the flexibility to begin with either syntactic or semantic mapping based on the specific case.
Data access	"*Take into consideration. Who will have access to data and who won't?*" [IP-2]<br><br>"*It may be worth describing to some degree is the fact that it also depends on who is doing the design and the implementation, because if you don't have access to the actual data then there are other challenges that you need to keep in mind*" [IP-6]<br><br>"*Define the roles and data access restriction.*" [QP-2]<br><br>"*It may be worth to note that the not all roles/people on the interdisciplinary team may have access to the patient-level data, and this may therefore need to be taken into account in the design of the process. For example, the ETL implementation team may use "dummy"/synthetic data for development and testing, and only people with data access may be able to execute the ETL on the actual data.*" [QP-6]	Incorporated data access as part of the Data Information layers of consideration. Since some developers do not have direct access to real data, it is essential to emphasize the use of synthetic or dummy data when access is restricted.
**Initiation**		
Intended use case	"*So, I think it's also important to note here that if the intention of the researchers to utilize standardized tools and methods as well as generate evidence as part of a network. Then you should be working with others in that network to make sure that you are all aligning on the same approaches for how you're standardizing your data. I think that's when it comes to cancer data in particular. I think that's very important because it can be up to interpretation. Sometimes, and we want to make sure when we are generating evidence that scale across multiple different data sources that the underlying assumptions are all the same, which is the point of data standardization is that we can standardize this underlying assumptions.*" [IP-10]	Added this detail as an important consideration during the harmonization process, emphasizing the need to define the intended use case clearly. It is essential to identify other projects that may align with the intended use case and ensure that all underlying assumptions are consistent and well-aligned. This alignment is crucial for ensuring scalable evidence generation.
Oncology extension	"*So technically there's no oncology extension anymore … In version 5.3 of the CDM there was a CDM and there was an oncology*	Removed information on the oncology extension since it has been integrated into the CDM as part of version 5.4.

Themes & Subthemes	Feedback	Refinement for the Proposed Process
	extension that you had to add separately. But, in 5.4 the current version, the Episode tables are part of the CDM, so there's technically no extension anymore.It's just it's part of the CDM." [IP-8]	Restructured the layers of consideration into three categories: data, infrastructure, and team. Reframed this as part of Infrastructure consideration layer
OHDSI ecosystem	"One thing is we didn't really use White Rabbits. and Rabbit In A Hat … the issue is that they are open source tools and not every IT department will allow you to just run open source tools on your data." [IP-8]	Added the consideration of site-specific permissions for open-source tool installation as it may affect the harmonization process. This is included as part of the Infrastructure layer in the revised framework.
Infrastructure	*"I think the only piece I see that is missing is infrastructure. How these tools get deployed and used is a huge part of making the harmonization effort successful, and each institution will have its own set of unique requirements for handling the data and associated software. Moreover, infrastructure configurations are often specific to each institution and can often present a major bottleneck at the outset of a harmonization project.*" [IP-1]<br><br>"*It's always a bit challenging because the the ecosystem is not just a CDM … Then of course you want to be able to run your scripts to run studies and yeah, it really depends on what kind of research environment you have, whether that works or not.*" [IP-8]	Emphasized infrastructure as a key consideration throughout the process. This aspect was briefly mentioned but now has greater visibility in the overall process framework as a stand-alone layer, alongside data and team considerations.
Interdisciplinary team	"*Define the roles*" [QP-2]<br><br>"*…corresponding responsibilities should be agreed upon as early as possible*." [QP-6]	Added more detailed information on defining roles and responsibilities within the interdisciplinary team activity. This ensures clear expectations and avoids ambiguity, especially in collaborative settings. This is now structured as a stand-alone consideration layer, referred to as team.
Training	"*I think that the lack of familiarity with the specific vocabularies is always an issue … So, you need your doctors to be aware of the of the dictionaries of the relevant dictionaries. This is a tricky part …Would you like to have a training module on your process?*" [IP-3]<br><br>"*You need to make sure that all the people involved have enough training to contribute … people need to be able to use this at the end.*" [IP-6]<br><br>"*Training should also be foreseen as a key part of the project, both for the people involved in the data harmonization process itself, and for those that will use the resulting OMOP CDM database instance and OHDSI tools.*" [QP-6]	Incorporated training into the team consideration layer. Training is critical to ensure participants are familiar with data sources, use cases, OMOP CDM, and relevant vocabularies to participate meaningfully in the harmonization process.
Specification & Profiling		
Suitable data sources	"*…with respect to identifying the most suitable data sources, usually what we find with our clients is that they've already identified those data sources.*" [IP-5]	Changed the activity of identifying suitable data sources from the "Specification & Profiling" phase to the "Initiation" step. The "Specification & Profiling" phase has been renamed to "Requirement Analysis" to more accurately reflect its broader focus on

Themes & Subthemes	Feedback	Refinement for the Proposed Process
		both data and infrastructure requirements.
Analysis data information	"*It's important to understand is a data set focused on one type of cancer or is it just patients with any malignancy because that might change how you approach it, what you're mapping, what you're looking. I think the next step is to look at what are those things that we can observe? … Is there anything that any potential data quality issues that could come up … Understand the type of data you have related to the type of cancer. And then if it is a particular type of cancer, what type of data elements are there? And then think through, OK, given these data elements. What potential issues might I observe that I would need to account for as I'm standardizing the data?*" [IP-10]	Added information to understand and verify the cancer type within the data sources, as this can impact the harmonization approach. For example, it may influence the development of transformation rules and the design of algorithms to derive specific information.
Data prioritization	"*Maybe it would be great if also the minimal dataset would be described what can be used for cancer studies (e.g. which diagnosis, measurements have to be present). Or what how to prioritize which data is converted first (this would be under specification and profiling task). As there can be many data points from different sources and with different quality then adding them will take time and there should be prioritization how data is converted to OMOP*." [QP-5]	The minimal dataset requirement is outside the scope of this thesis, as it should be defined by organizations or relevant standards. Data prioritization is already included as part of the process, but this will be more explicitly highlighted in each phase.
Infrastructure assessment	"*The infrastructure needs, technology choices … should be agreed upon as early as possible*." [QP-6]	Added infrastructure assessment and technology choices as new activities in the proposed process to ensure these decisions are made early and integrated into the planning phases.
**Design Plan**		
Synthetic data	"*In some way missing in the documentation how you'll get the synthetic data, how you generate it to be, you know, compatible with the real one*." [IP-2]<br><br>"*More details about the proposed synthetic data. How these will be created to reflect the real data plausibly.*" [QP-2]	Refined the synthetic data section to consider how synthetic data should be generated and how its compatibility with real data is ensured, especially where real data access is restricted.
Mapping issues	"*A strategy what to do when some required data elements (e.g. imaging) won't be transformable into OMOP CDM.*" [QP-2]<br><br>"*Give more insights for troubleshooting (especially for cases where no appropriate mapping/vocabulary is available)*" [QP-7]	In addition to the strategy outlined in the initial version, which follows the THEMIS convention and OMOP Oncology guidelines, there is now a highlighted emphasis on the need for active communication with relevant OHDSI working groups to resolve mapping issues. Temporary solutions, such as creating custom mappings or using concept_ID = 0, should also be considered when appropriate.
Modelling cancer information	"*So, I'm just so one thing I will say is like you have it very nicely mapped here (the modelling cancer information), but it's not always that clean … It's not gonna all go to those exact tables. It's actually up to the vocabulary domain … It's important to make sure that it's really the domain of the vocabulary that's driving the movement*." [IP-10]	Refined the section on modeling cancer information.

Themes & Subthemes	Feedback	Refinement for the Proposed Process
	"*On pages 9 and 10, it may be worth to indicate that, since the cancer diagnosis can be done using different methods, the cancer diagnosis can also be coded using standard terminologies other than ICD-O-3, such as SNOMED. Similarly, the procedures can be coded using standard concepts from several terminologies, including SNOMED, ICD10PCS, CPT4, etc.*" [QP-6]	
**Technical Implementation**		
Data curation	"*The only part that I don't fully understand actually the data curation part. What I often find is that these sorts of processes are a little bit more intertwined … But it does make sense to actually have it there. I think it's a nice addition … Maybe before the extraction part, at least in my head, it's usually more the data scientists that curate the data and then it's the data engineers that extract, transform and load the data. Because the engineers usually do not have the clinical or the scientific expert.*" [IP-5]<br><br>"*I think most of the data curation involved would happen before or as part of the extraction … I guess there are different types of data curation, right? Or, so if you can look at it as a preprocessing step, then indeed, much of that can be done before you actually start to process the extraction process … When you need to do some of the data curation, obviously as part of the process, that we usually do it, it looks a bit like what you have here.*" [IP-6]<br><br>"*The data curation, there's a lot involved there that we can't handle up front … Like data cleaning, there's a lot just involved in that one phrase. There you know deidentification depending on you know what you have in your data can be pretty challenging.So, each one of those pieces and data curation kind of like its own method into itself and can be difficult*" [IP-10]	Changed "data curation" to "data preprocessing" and repositioned it before the ETL phase. This adjustment aligns with real-world practices, where data is typically preprocessed prior to extraction. However, "data curation" is still referenced, as it may remain relevant throughout the process, depending on the use case. The term covers a broad range of topics, each with its own methodologies.
Transform	"*I would take Usagi, Athena and I'm not familiar with (KOIOS), but I assume they're also on the same line. I would take them out of the technical implementation. I'd rather make them part of the design plan … performing a transformation is something that is done using either SQL or what we use a specific Python packages that allow us to write SQL in Python.*" [IP-5]	Moved semantic and syntactic mapping tools to the Design Plan phase. Refined the Transform activity to include transformations using SQL, R, or Python to better reflect current industry practices.
**Maintenance**		
Update frequency plan	"*It's good to take into consideration how frequently you'll get data update. And how frequently? These data update changes in its structure and in its semantic over time.*" [IP-2]<br><br>"*The maintenance, it's actually rather critical to also plan that out from the beginning … The maintenance aspect is often overlooked, or at least not prioritized enough.*" [IP-6]	Added a data update frequency plan to the process, ensuring that maintenance considerations are incorporated from the beginning. This will help address the evolution of the data and its semantics over time.

Supplementary Table 5 | Academic expert feedback and refinements on the final evaluation

Themes & Subthemes	Feedback	Refinement for the Proposed Process
**Requirement Analysis**		
Risk identification & mitigation	*" Which strategies are available for risk identification and mitigation?"*	A dedicated activity for risk identification and mitigation was introduced, which was previously embedded within other activities. The revision highlights the importance of proactively identifying potential risks and considering mitigation strategies early in the process, while maintaining flexibility to adapt the approach based on the specific context of each environment.
**Maintenance**		
Optimization	*"…related to the life cycle of the process. Is it correct to think about "optimization" as an activity in the maintenance phase? In industry there are different types of maintenance: corrective, preventive, predictive, and proactive maintenance"*	Reframed the activity as "Periodic Review and Update for Optimization", emphasizing optimization as a key objective. This activity may involve various types of maintenance, such as corrective, preventive, or proactive, depending on specific needs and considerations within the process lifecycle.

Supplementary Table 6 | Detailed key steps of the proposed cancer harmonization process into OMOP CDM

Steps	Activities
**1. Initiation**	***Objective:*** Lay the groundwork by assembling the team, defining the intended use case for harmonization, and familiarizing with OHDSI ecosystem and related infrastructures.
**a. Team Aspects**	**a.i. Assemble an interdisciplinary team**<br>Form an interdisciplinary team that includes, but is not limited to:<br>• Medical experts (e.g., oncologists, doctors, nurses, pharmacists)<br>• Data experts (e.g., data owners, providers, stewards)<br>• ETL experts (e.g., data engineers, data analysts, software developers)<br>• OMOP CDM experts with oncology-specialized knowledge<br>• Health informaticians<br>Each team member's roles and responsibilities must be clearly defined to ensure clear expectations and avoids ambiguity, as the process is iterative and resource intensive.<br><br>**a.ii. Training**<br>Provide necessary training to team members unfamiliar with the harmonization process, OMOP CDM, vocabulary codes, OHDSI tools, and technical implementation. This training may cover harmonization steps, roles, responsibilities, medical and technical terminology, OMOP CDM structure and standardized vocabularies, and usage of OHDSI tools based on each team member's involvement.
**b. Data Aspects**	**b.i. Define the intended use case**<br>The harmonization process begins by clearly defining the intended use case, including outlining its objectives and establishing inclusion and exclusion criteria.<br><br>**b.ii. Identify prioritized key variables**<br>Essential data elements such as cancer type, treatment details, study period, and minimum required data volume must be identified early to guide the harmonization process along with its prioritization for harmonization.<br><br>**b.iii. Identify similar projects**<br>In addition, it is essential to identify other projects that may align with the intended use case to ensure that all underlying assumptions during the harmonization process are consistent and well-aligned across different projects. This alignment is crucial for ensuring scalable evidence generation.<br><br>**b.iv. Identify suitable data sources**<br>This step involves specifying data source(s) that will be harmonized, either using single or multiple data sources, based on the intended use case, and alignment with the research questions. Data source(s) compatibility with OMOP CDM should be assessed, considering that OMOP is still evolving and may not support all data types.
**c. Infrastructure Aspects**	**c.i. Familiarize with the OHDSI ecosystem**<br>In this activity, the focus is on exploration and familiarization with the OHDSI ecosystem, including OMOP CDM, standardized vocabularies, and relevant tools & algorithms. This step provides the team with a solid foundation in the tools and frameworks essential for harmonizing cancer data.<br><br>**c.ii. Institution rules and regulation understanding**<br>Understanding site-specific rules and regulations for infrastructure is crucial, as these will directly influence the harmonization approach. This includes identifying any technical constraints, limitations imposed by the institution, and permissions regarding the installation of open-source tools. This early awareness helps in planning for potential obstacles and ensures the harmonization approach is aligned with institutional policies.
**2. Requirement Analysis**	***Objective:*** Assess detailed requirements for team expertise, data, and infrastructure to identify potential challenges or gaps, along with corresponding mitigation strategies.<br><br>This step involves understanding the needs for each aspect, team, data, and infrastructure, and identifying potential issues early. By collaborating and analyzing the available resources, the team can prioritize tasks and define risk mitigation strategies. This ensures smooth progress despite constraints such as limited time or resources.

Steps	Activities
**a. Data Aspects**	**a.i. Data information analysis**<br>Analyze the characteristics of the data sources to guide the harmonization process, identify specific actions, and detect potential issues. This analysis includes assessing the data sources, content, structure, model, quality, and access. A key aspect of this analysis is the verification of the presence of essential variables, particularly cancer type, as it directly influences the harmonization approach. Understanding the cancer type within the data sources is critical as each cancer type is unique and complex and it may affect the development of transformation rules and the design of algorithms for the harmonization process.<br>Certain cancer-related data may not be directly available in the source data, such as drug regimens, cancer staging, and disease progression. These variables often need to be derived through data abstraction, utilizing specialized algorithms, scripts, or existing tools developed by the OHDSI community.<br><br>**a.ii. Semantic vocabulary and syntactic structure analysis**<br>• **Semantic vocabulary analysis**<br>Evaluate the vocabulary along with its domain coverage used in the data source to ensure proper alignment with OMOP standardized concepts. If non-standard vocabularies or local system codes are used, prepare a local dictionary to facilitate mapping and mitigate ambiguity. If the source data uses country-specific language not supported by OMOP CDM, a translation may be required before mapping.<br><br>• **Syntactic structure analysis**<br>The table structures of the source data should be analyzed to understand the relationships between tables, field formats, and the need for modifications to align with OMOP CDM's person-centric structure. Some data sources might need to be restructured (e.g., from wide table format and traverse it to long format) for easier harmonization process and to conform with OMOP standards. The goal is to ensure that the source data can be transformed seamlessly into OMOP CDM tables<br><br>**Potential Tools:** White Rabbit for data profiling.<br><br>**a.iii. Post-Analysis evaluation**<br>Key variables identified within data source should be validated by medical experts, ensuring that they align with the intended use case. Descriptive statistics should be generated to assess the frequency and distribution of the data, which will be useful for evaluating the completeness of the transformed data. The interdisciplinary team should evaluate whether data preprocessing or custom algorithms are required to handle specific data characteristics.<br>**a.iv Risk identification & mitigation**<br>This activity focuses on identifying potential risks related to data quality and handling, along with establishing agreed-upon mitigation strategies. Common risks include information loss during the harmonization process, unmapped concepts, issues with data provenance, redundancy, discrepancies, and other data quality challenges that may arise throughout the harmonization process. Risk identification is a critical activity that must be addressed early in the process. Mitigation strategies should align with established THEMIS convention and OHDSI Oncology guidelines and be tailored to the specific use case and the infrastructure environment. All strategies must be agreed upon by the team and thoroughly documented.
**b. Infrastructure Aspects**	**b.i. Infrastructure assessment**<br>Conduct a thorough assessment of the research environment and available institutional infrastructure. Identify the existing technical tools, resources, and capabilities, as well as any limitations that might affect the harmonization process. This involves identifying infrastructure needs such as computing resources, database servers, and network access. Review specific resources like cloud storage, computational power, and security protocols. Assess whether the current infrastructure can support the data volume and complexity expected in the project. The analysis should also consider existing constraints and limitations that might affect harmonization, such as system compatibility. Early decisions on infrastructure will ensure smooth planning and execution. Ensure that the chosen technologies are compatible with the facility's rules and guidelines and documented thoroughly.

Steps	Activities
	**b.ii. Risk identification & mitigation**<br>This activity involves identifying any potential risks that could impact the harmonization process. These risks may include inadequate infrastructure, limited technical capabilities, timeline constraints, infrastructure-specific regulatory limitations that may restrict the use of open-source tools, and potential risks associated with adopting specific ETL approaches. Early identification of these risks enables proactive planning and the formulation of mitigation strategies. The mitigation approach is adapted to the specific context, agreed upon by the team, and properly documented.
**c. Team Aspects**	**c.i. Risk identification & mitigation**<br>Following the initial team formation, based on the intended use case and infrastructure availability, it is important to reassess whether the team possesses the necessary expertise to support the harmonization process. This includes identifying any potential skill gaps, associated risks, and corresponding mitigation strategies. Where gaps are found, additional expertise may be considered to ensure all critical areas are covered. Conducting this assessment early helps ensure a seamless process. The strategies adopted should be tailored to the specific context, agreed upon by all team members, and clearly documented.
**3. Design Plan**	***Objective:*** Develop harmonization strategies, define transformation rules, plan semantic and structural mappings, and establish evaluation and maintenance strategies.<br><br>The design plan phase focuses on developing comprehensive harmonization strategies, covering data preprocessing, curation, ETL logic, and maintenance to guide the technical implementation. During this phase, no actual transformation is done yet; rather, the strategies are mapped out. The design plan is developed iteratively through interdisciplinary discussions to ensure accuracy, consistency, and minimal data loss.<br>It may also be beneficial to utilize synthetic or dummy dataset to test the harmonization logic, algorithms, or transformation scripts before full-scale implementation. This allows the team to address potential issues early in the process.<br>For maintenance strategies, a data update frequency plan is essential, ensuring that maintenance considerations are incorporated from the beginning. This will help address the evolution of the data and its semantics over time as well as account for updates to OMOP CDM and the OHDSI ecosystem.
**a. Data Aspects**	**a.i. Develop data mapping strategies**<br>The design plan focuses on mapping the agreed prioritized data to ensure alignment with OMOP CDM standards. Define transformation rules with agreed conventions within the team, adhering THEMIS conventions & OMOP Oncology guidelines. If essential oncology-related information in data sources needed data abstraction, such as drug regimens, identify appropriate OHDSI tools or custom algorithms to extract or derive the necessary data. Tools like ARTEMIS or OncoRegimenFinder, can be used to address data abstraction needs for treatment regimen. These tools should be tested for compatibility using either a subset of the data sources or synthetic/dummy datasets.<br>In addition, potential issues and challenges in mapping should be identified, and solutions should be developed, and the entire process should be thoroughly documented. Both PROs and imaging data currently lack well-established conventions within OMOP CDM, and their representation is under continuous development. Therefore, interdisciplinary discussions should determine a temporary mapping strategy, and engagement with the OHDSI community is recommended for alignment with ongoing standardization efforts. Furthermore, develop the evaluation and maintenance plan that will be conducted.<br><br>**a.ii. Semantic vocabulary and syntactic structure mapping**<br>• **Semantic vocabulary mapping**<br>Semantic mapping ensures that local or source codes are mapped to OMOP standardized vocabularies. Prioritize the most frequently used terms in the data sources for efficient mapping. Utilize tools like Athena, Usagi, and KOIOS for mapping to OMOP Standardized Vocabularies.<br>If locally developed codes are used, a local code dictionary should be used for reference when doing the mapping to prevent ambiguity in mapping. Disagreements on local term interpretation should be resolved through collaboration and discussion with medical and data experts. If the source data uses country-specific languages that are not supported within OMOP CDM, translation may be required before

Steps	Activities
	mapping. If none of the above strategies suffice, consultation with other relevant projects or OHDSI workgroups for better solution alignment assumptions is advised.<br>**Potential Tools:** Athena, Usagi, and KOIOS.<br><br>• **Syntactic structure mapping**<br>Structural mapping aligns data elements from the source data to OMOP CDM tables based on domain attributes and ensure alignment with OHDSI guidelines. Ensure proper linkage of oncology-related events, such as treatment regimens and disease progression, within the Episode and Episode_Event tables. Collaborate with OMOP CDM experts to ensure accurate mapping to the appropriate tables.<br><br>**Potential Tools:** Rabbit In A Hat for documenting ETL logic and mapping relationships between source and OMOP CDM tables.<br><br>**a.iii. Strategy Evaluation**<br>The mapping strategy must undergo a thorough review by expert teams to ensure its completeness, accuracy, and alignment with OMOP conventions, while minimizing information loss. It is crucial to verify that all prioritized cancer-specific data elements are adequately accounted for. Furthermore, key evaluation metrics should be identified to assess the completeness of the mapping process. Additionally, the ETL logic should be rigorously evaluated through unit tests to ensure that all potential data quality issues are addressed and that the logic functions as intended.<br><br>**Potential Tools:** Rabbit In A Hat for creating a unit test framework
**b. Infrastructure Aspects**	**b.i. Develop infrastructure plan for harmonization and maintenance**<br>Develop a comprehensive technical infrastructure plan based on findings from the Requirement Analysis phase. Review any identified gaps or issues and address them with solutions or adjustments to the infrastructure. Ensure that the infrastructure plan accommodates scalability to handle large oncology datasets, meets the needs of the project, and supports maintenance. The infrastructure plan should also consider compliance with data access policies and privacy regulations. If external parties are involved in the harmonization process, ensure that the infrastructure supports secure data access and confidentiality requirements. Set up mechanisms for data anonymization or use of synthetic datasets to comply with privacy laws.<br><br>**b.ii. Review chosen tools and technologies compabilities**<br>Assess the compatibility and interoperability of chosen tools, infrastructure, and technologies with OMOP CDM standards and will support the technical requirements for the data transformation. This activity is a key step to ensure that there are no last-minute compatibility issues during the implementation phase.<br><br>**b.iii. Set up testing and evaluation environment**<br>Set up testing environments to simulate real-world data transformations before full-scale implementation. This includes setting up synthetic or dummy datasets to evaluate the transformation logic and ensure it works as expected. Ensure that all technical aspects are tested in a controlled environment to identify and resolve potential issues early in the process. The testing environment should be capable of running unit tests, validation checks, and quality assurance tasks to ensure the transformation process is reliable and accurate.
**c. Team Aspects**	**c.i. Review harmonization and maintenance strategies**<br>All team members should review and discuss the harmonization and maintenance strategies. Ensure all decisions are thoroughly documented for references and to track any changes. This includes identifying potential implementation issues and disruptions (e.g., data quality issues, inconsistencies, missing values, unmapped concepts, technical constraints). The team should plan for mitigation strategies to address these issues before actual implementation. Specific areas of focus include:<br>• Semantic & Structural Mapping Strategies: Ensure these align with THEMIS conventions and OMOP Oncology Guidelines.<br>• Transformation Rules & Logic: Verify that the transformation rules are well-defined and aligned with the use case.<br>• Evaluation Plan: Review the evaluation strategies to ensure accurate representation of cancer data into OMOP CDM

Steps	Activities
	• Infrastructure Plan: Review the technology choices and confirm alignment with scalability for future data or research updates.<br>• Maintenance Plan: Review the strategy for long-term data maintenance and handling evolving research needs.
**4. Technical Implementation**	***Objective:*** Execute the harmonization process, ensure quality control, and validate data transformation.<br><br>Technical implementation is a resource extensive steps requiring a lot of coding. During implementation, errors and inconsistencies should be addressed through iterative testing and debugging. Given the complexity of data transformation, OMOP CDM adoption should be viewed as an ongoing process rather than a one-time task.
**a. Infrastructure Aspects**	**a.i. Technical setup**<br>Once the infrastructure plan is finalized, the technical components required for the harmonization process are set up. Ensure that the infrastructure is fully operational and optimized to facilitate the harmonization process and that the necessary OMOP CDM integration tools (e.g., OHDSI ecosystem tools) are properly configured. Verify that the infrastructure supports the technical requirements for data transformation and is aligned with the latest updates in OMOP CDM and related vocabularies.<br>Execute the data transformation based on the designed plan, ensuring that data is curated, extracted, transformed, and loaded into OMOP CDM as per the established rules and frameworks. During this phase, infrastructure should be continuously monitored to detect and address any issues such as system overloads, performance issues, or data integration problems. If any technical problems arise, they should be addressed promptly to avoid delays.
**b. Team Aspects**	**b.i. Execute harmonization strategies**<br>Focus on executing the harmonization plan while ensuring quality control and validation, ensuring that data is pre-processed, curated, transformed, and loaded into OMOP CDM as planned. This include review and validate transformed data for clinical accuracy and relevance, address any issues or errors that arise during the transformation process, and ensure all decisions and adjustment made during the implementation are thoroughly documented for future reference.
**c. Data Aspects**	**c.i. Data preprocessing & curation**<br>Data preprocessing and curation might be needed for some data sources depending on the use case. Data curation can be performed throughout the harmonization process, as it may be needed at different stages.<br>Data preprocessing & data curation include, but not limited to:<br>• Restructuring data format (e.g., to fit OMOP CDM's person-centric structure, traverse wide-format into long-format data)<br>• Data cleaning, deduplication, handling missing values, filtering and removing inconsistent or erroneous records<br>• Merging multiple sources, handling discrepancies & redundant information, and addressing varying level of granularities across data sources<br>• De-identification for privacy compliance<br>• Translation of local terminologies where necessary<br>• Cancer data abstraction (e.g., extracting treatment regimens)<br>• Extracting and restructuring free-text data using rule-based approaches, parsing techniques, regular expressions (RegEx), or other NLP-based algorithms.<br>• Model-specific data extraction (e.g., if the data sources use specific data model, such as HL7 FHIR or openEHR, corresponding tools or algorithms may be required for extraction)<br><br>**Potential Tools**: SQL, Python, R, RegEx for data preprcessing & data curation. ARTEMIS and OncoRegimenFinder algorithm for extracting treatment regimen.<br><br>**c.ii. Preprocessing & curation evaluation**<br>Validate the accuracy of the data after preprocessing, ensuring that translations and curation steps have been correctly applied and identify potential information lost.<br><br>**c.iii. Extract-Transform-Load (ETL)**<br>The processed and curated data is then extracted and transformed into OMOP CDM format. Extracted data should be validated for completeness and correctness. Tools like SQL, R, or Python can be used to implement the transformation logic. During this phase,

Steps	Activities
	ensure that all data is correctly loaded into OMOP CDM, adhering to the transformation rules defined in the design plan.<br><br>**Potential Tools:** SQL, R, Python.<br>**c.iv. Transformed Data Evaluation**<br>Ensuring the accuracy and reliability of data transformation into OMOP CDM requires a comprehensive evaluation framework. The evaluation process is iterative, requiring continuous testing, debugging, and refinement until all quality checks are satisfactorily passed. This evaluation includes, but is not limited to:<br><br>***c.iv.1. Mapping Validation with Experts (Semantic and Structural)***<br>Accurate mapping of source data to OMOP CDM requires rigorous validation of both semantic and structural mappings:<br>• Semantic validation ensures correct mapping to standardized terminologies.<br>• Structural validation ensures that the mapped concepts are appropriately placed into OMOP CDM tables while preserving logical relationships.<br>• Mapping coverage is assessed by measuring the percentage of mapped codes and records and evaluating where mapping may have lost detail or was infeasible.<br><br>***c.iv.2. Data Quality Checks***<br>To ensure data quality, various quality control tools such as Achilles, Data Quality Dashboard (DQD), and ARES can be deployed:<br>• Comparing transformed data with the original data sources is a critical step for quality assessment. Demographic breakdowns before and after transformation should be examined to detect any shifts, inconsistencies, or potential information loss. Achilles can facilitate this process by providing an overview of the transformed data, characterizing key attributes such as demographics and clinical event distributions, helping to identify any discrepancies between the original and transformed datasets.<br>• DQD is used to assess conformance, completeness, and plausibility by running automated data quality tests.<br>• ARES identifies mapping coverage gaps and source-specific quality issues. Utilized ARES for comparing multiple data sources, ensuring consistency across network-wide studies<br>Errors, warnings, or inconsistencies must be iteratively addressed through ETL code refinement or updated mappings until the data meets quality standards.<br><br>***c.iv.3. Empirical Model Testing (Use-Case Validation)***<br>To assess the effectiveness of OMOP conversion for real-world research applications, empirical model testing is conducted.<br>• Cancer cohort validation is conducted in ATLAS to ensure the transformed data reflects expected clinical trends. Cohorts are defined using specific inclusion criteria, such as metastatic cancer, cancers with genetic variations, lymph node involvement, cancer staging, and survival outcomes.<br>• Custom test queries are executed to verify the syntactic and semantic integrity of the transformed data. These tests help identify discrepancies and enable iterative debugging.<br><br>**Potential Tools:** Rabbit In A Hat for creating unit test framework, Achilles, Data Quality Dashboard (DQD), ARES, and ATLAS.
**5. Maintenance**<br><br><br><br><br><br><br><br>**a. Team Aspects**	***Objective:*** Ensure continuous monitoring, updating, and maintenance of the transformed data for optimization.<br><br>Transforming data into OMOP CDM is not a one-time process but an ongoing effort that requires regular updates and refinements. As data sources, OMOP CDM, vocabularies, and OHDSI tools evolve, depending on the intended use case, it is essential to periodically review and update mappings and transformation rules.<br><br>**a.i. Post-Transformation Training**<br>In some cases, external parties may be responsible for conducting the harmonization process. In these instances, it is essential to provide appropriate training and handover to internal teams on how to utilize the OHDSI ecosystem, associated infrastructures, and manage the transformed data. This ensures that users can effectively apply the OMOP CDM in accordance with the intended use case. Additionally, periodic training

Steps	Activities
	should be conducted to maintain up-to-date knowledge and ensure continuous improvement.<br><br>**a.ii. Ongoing team engagement**<br>A dedicated team should oversee the long-term administration of OMOP CDM, ensuring that updates, vocabulary changes, emerging data requirements, and modifications in infrastructure are properly accommodated over time.<br>Continuous engagement with medical experts, data experts, technical experts, OMOP CDM professionals, and OHDSI community is crucial for maintaining high data quality and clinical relevance. This collaborative approach ensures that the transformed data remains accurate, standardized, and valuable for ongoing research and analytics across different domains.
**b. Data Aspects**	**b.i. Periodic review and update for optimization**<br>Data sources and their associated content, such as cancer-related information, semantic vocabulary, and syntactic structure, may evolve over time. Depending on the specific use case, periodic reviews and updates of the data sources may be necessary to ensure that the harmonized data remains relevant and up-to-date. These updates may include revisiting mapping strategies to incorporate new or modified data elements. This optimization activity includes but not limited to:<br>• Preventive maintenance: conducting scheduled reviews (e.g., vocabulary updates every six months) to preemptively adjust for known or expected changes.<br>• Corrective maintenance: addressing identified misalignments or errors post-harmonization.<br>• Proactive maintenance: systematically improving mapping quality and data relevance beyond just fixing issues, such as refining data transformation logic based on long-term goals or anticipated future data changes.<br><br>The frequency and depth of these updates should be based on the specific use case, and all updates must be documented and agreed upon by the team.
**c. Infrastructure Aspects**	**c.i. Periodic review and update for optimization**<br>The interdependencies between various tools and infrastructure components should be carefully managed to prevent disruptions. It is essential to regularly review and optimized the infrastructure to ensure it remains robust, scalable and reliable to evolving data requirements, technologies, OHDSI ecosystem, or additional research needs. This may include the following activity but not limited to:<br>• Preventive maintenance: scheduled checks and updates to infrastructure components (e.g., regular system and security upgrades) to avoid potential failures.<br>• Corrective maintenance: fixing issues after they occur, such as restoring failed actions or correcting system misconfigurations.<br>• Proactive maintenance: strategically upgrading infrastructure or tools in anticipation of future demands (e.g., scaling systems to handle new data sources or integrating new OHDSI ecosystem).<br><br>These efforts ensure the harmonization process remains adaptable and efficient as project needs evolve. Maintenance should be tailored to the specific research environment.

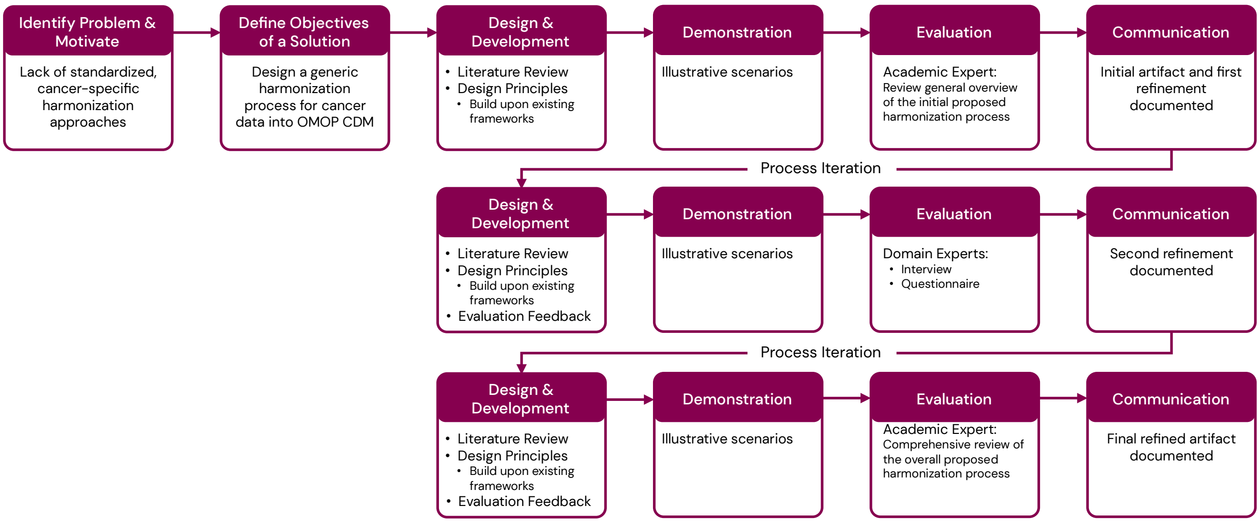

Supplementary Fig. 2 | DSRM framework process as applied in this study with three iterations

Supplementary Table 7 | Search terms in PubMed, CINAHL, Embase, WoS, ACM, IEEE, and Google Scholar databases

Database	Search	Search String	Results
PubMed	#1	("neoplasms"[MeSH Terms] OR ("adeno*"[All Fields] OR "angiosarcoma*"[All Fields] OR "astrocytoma*"[All Fields] OR "blastoma*"[All Fields] OR "burkitt*"[All Fields] OR "cancer*"[All Fields] OR "carcino*"[All Fields] OR "cholangiocarcinoma*"[All Fields] OR "chondrosarcoma*"[All Fields] OR "chordoma*"[All Fields] OR "choriocarcinoma*"[All Fields] OR "chronic myeloproliferative disorder*"[All Fields] OR "craniopharyngioma*"[All Fields] OR "cytoma*"[All Fields] OR "ependymoma*"[All Fields] OR "esthesioneuroblastoma*"[All Fields] OR "fibrosarcoma*"[All Fields] OR "germinoma*"[All Fields] OR "gestational trophoblastic disease*"[All Fields] OR "glioblastoma*"[All Fields] OR "glioma*"[All Fields] OR "gonadoblastoma*"[All Fields] OR "hemangiosarcoma*"[All Fields] OR "hepatoblastoma*"[All Fields] OR "histiocyto*"[All Fields] OR "hodgkin*"[All Fields] OR "leukaemi*"[All Fields] OR "leukemi*"[All Fields] OR "linitis plastica"[All Fields] OR "lymphangio*"[All Fields] OR "lympho*"[All Fields] OR "macroglobulinemia*"[All Fields] OR "malignan*"[All Fields] OR "medulloblastoma*"[All Fields] OR "melanoma*"[All Fields] OR "meningioma*"[All Fields] OR "mesenchymoma*"[All Fields] OR "mesone*"[All Fields] OR "mesothelioma*"[All Fields] OR "metasta*"[All Fields] OR "mycosis fungoid*"[All Fields] OR "myelo*"[All Fields] OR "Myelodysplastic Syndromes"[All Fields] OR "neoplas*"[All Fields] OR "neuroblastoma*"[All Fields] OR "neuroma*"[All Fields] OR "nonmelanoma*"[All Fields] OR "nonseminoma*"[All Fields] OR "osteosarcoma*"[All Fields] OR "pancreatoblastoma*"[All Fields] OR "papillomatos*"[All Fields] OR "paraganglioma*"[All Fields] OR "paraneoplas*"[All Fields] OR "Persistent Trophoblastic Disease"[All Fields] OR "phaeochromocytoma*"[All Fields] OR "pheochromocytoma*"[All Fields] OR "pineoblastoma*"[All Fields] OR "plasmacytoma*"[All Fields] OR "Polycythaemia Vera"[All Fields] OR "Pseudomyxoma Peritonei"[All Fields] OR "rhabdomyosarcoma*"[All Fields] OR "retinoblastoma*"[All Fields] OR "rhabdo*"[All Fields] OR "sarcoma*"[All Fields] OR "schwannoma*"[All Fields] OR "seminoma*"[All Fields] OR "sezary syndrome*"[All Fields] OR "terato*"[All Fields] OR "thrombocythaemia*"[All Fields] OR "tumor*"[All Fields] OR "tumour*"[All Fields])) AND 2014/01/01:2025/02/28[Date - Publication] AND "English"[Language]	3,000,828
	#2	"omop*"[All Fields] OR "Observational Medical Outcomes Partnership"[All Fields] OR "OHDSI"[All Fields] OR "Observational Health Data Sciences and Informatics"[All Fields]) AND 2014/01/01:2025/02/28[Date - Publication] AND "English"[Language]	671
	#3	#1 AND #2	109
CINAHL	S1	(MH "Neoplasms+") Publication Date: 20140101-20250231; English Language	360,401
	S2	adeno* OR angiosarcoma* OR astrocytoma* OR blastoma* OR burkitt* OR cancer* OR carcino* OR cholangiocarcinoma* OR chondrosarcoma* OR chordoma* OR choriocarcinoma* OR "Chronic Myeloproliferative Disorder*" OR craniopharyngioma* OR cytoma* OR ependymoma* OR esthesioneuroblastoma* OR fibrosarcoma* OR germinoma* OR "Gestational Trophoblastic Disease*" OR glioblastoma* OR glioma* OR gonadoblastoma* OR hemangiosarcoma* OR hepatoblastoma* OR histiocyto* OR hodgkin* OR leukaemi* OR leukemi* OR "linitis plastica" OR lymphangio* OR lympho* OR macroglobulinemia* OR malignan* OR medulloblastoma* OR melanoma* OR meningioma* OR mesenchymoma* OR mesone* OR mesothelioma* OR metasta* OR "Mycosis Fungoid*" OR myelo* OR "Myelodysplastic Syndromes" OR neoplas* OR neuroblastoma* OR neuroma* OR nonmelanoma* OR nonseminoma* OR osteosarcoma* OR pancreatoblastoma* OR papillomatos* OR paraganglioma* OR paraneoplas* OR "Persistent Trophoblastic Disease" OR phaeochromocytoma* OR pheochromocytoma* OR pineoblastoma* OR plasmacytoma* OR "Polycythaemia Vera" OR "Pseudomyxoma Peritonei" OR rhabdomyosarcoma* OR retinoblastoma* OR rhabdo* or sarcoma* OR schwannoma* OR seminoma* OR "Sézary Syndrome*" OR	641,937

Database	Search	Search String	Results
		terato* OR thrombocythaemia* OR tumor* OR tumour* Publication Date: 20140101-20250231; English Language	
	S3	S1 OR S2	653,759
	S4	OMOP* OR "Observational Medical Outcomes Partnership" OR OHDSI OR "Observational Health Data Sciences and Informatics" Publication Date: 20140101-20250231; English Language	213
	S5	S3 AND S4	23
Embase	#1	'neoplasm'/exp AND [english]/lim AND [01-01-2014]/sd NOT [01-03-2025]/sd	3,178,754
	#2	(adeno* OR angiosarcoma* OR astrocytoma* OR blastoma* OR burkitt* OR cancer* OR carcino* OR cholangiocarcinoma* OR chondrosarcoma* OR chordoma* OR choriocarcinoma* OR 'chronic myeloproliferative disorder*' OR craniopharyngioma* OR cytoma* OR ependymoma* OR esthesioneuroblastoma* OR fibrosarcoma* OR germinoma* OR 'gestational trophoblastic disease*' OR glioblastoma* OR glioma* OR gonadoblastoma* OR hemangiosarcoma* OR hepatoblastoma* OR histiocyto* OR hodgkin* OR leukaemi* OR leukemi* OR 'linitis plastica' OR lymphangio* OR lympho* OR macroglobulinemia* OR malignan* OR medulloblastoma* OR melanoma* OR meningioma* OR mesenchymoma* OR mesone* OR mesothelioma* OR metasta* OR 'mycosis fungoid*' OR myelo* OR 'myelodysplastic syndromes' OR neoplas* OR neuroblastoma* OR neuroma* OR nonmelanoma* OR nonseminoma* OR osteosarcoma* OR pancreatoblastoma* OR papillomatos* OR paraganglioma* OR paraneoplas* OR 'persistent trophoblastic disease' OR phaeochromocytoma* OR pheochromocytoma* OR pineoblastoma* OR plasmacytoma* OR 'polycythaemia vera' OR 'pseudomyxoma peritonei' OR rhabdomyosarcoma* OR retinoblastoma* OR rhabdo* OR sarcoma* OR schwannoma* OR seminoma* OR 'sézary syndrome*' OR terato* OR thrombocythaemia* OR tumor* OR tumour*) AND [english]/lim AND [01-01-2014]/sd NOT [01-03-2025]/sd	4,962,977
	#3	#1 OR #2	5,073,153
	#4	(omop* OR 'observational medical outcomes partnership' OR ohdsi OR 'observational health data sciences and informatics') AND [english]/lim AND [01-01-2014]/sd NOT [01-03-2025]/sd	940
	#5	#3 AND #4	213
WoS	S1	(adeno* OR angiosarcoma* OR astrocytoma* OR blastoma* OR cancer* OR carcino* OR chordoma* OR craniopharyngioma* OR cytoma* OR ependymoma* OR esthesioneuroblastoma* OR "Trophoblastic Disease*" OR glioblastoma* OR glioma* OR gonadoblastoma* OR hepatoblastoma* OR histiocyto* OR leukaemi* OR leukemi* OR lymphangio* OR lympho* OR macroglobulinemia* OR malignan* OR melanoma* OR meningioma* OR mesenchymoma* OR mesone* OR mesothelioma* OR metasta* OR "Mycosis Fungoid*" OR myelo* OR neoplas* OR neuroblastoma* OR osteosarcoma* OR papillomatos* OR paraganglioma* OR paraneoplas* OR phaeochromocytoma* OR pheochromocytoma* OR plasmacytoma* OR retinoblastoma* OR rhabdo* or sarcoma* OR "Sézary Syndrome*" OR terato* OR tumor* OR tumour*) (All Fields) AND (OMOP* OR "Observational Medical Outcomes Partnership" OR OHDSI OR "Observational Health Data Sciences and Informatics") (All Fields) AND English (Language) | Timespan: 2014-01-01 to 2025-02-28 (Publication Date)	90
ACM Digital Library	#1	[[All: adeno*] OR [All: angiosarcoma*] OR [All: astrocytoma*] OR [All: blastoma*] OR [All: burkitt*] OR [All: cancer*] OR [All: carcino*] OR [All: cholangiocarcinoma*] OR [All: chondrosarcoma*] OR [All: chordoma*] OR [All: choriocarcinoma*] OR [All: "chronic myeloproliferative disorder*"] OR [All: craniopharyngioma*] OR [All: cytoma*] OR [All: ependymoma*] OR [All: esthesioneuroblastoma*] OR [All: fibrosarcoma*] OR [All: germinoma*] OR [All: "gestational trophoblastic disease*"] OR [All: glioblastoma*] OR [All: glioma*] OR [All: gonadoblastoma*] OR [All: hemangiosarcoma*] OR [All: hepatoblastoma*] OR [All: histiocyto*] OR [All: hodgkin*] OR [All: leukaemi*] OR [All: leukemi*] OR [All: "linitis plastica"] OR [All: lymphangio*] OR [All: lympho*] OR [All: macroglobulinemia*] OR [All: malignan*] OR [All: medulloblastoma*] OR [All: melanoma*] OR [All: meningioma*] OR [All: mesenchymoma*] OR [All: mesone*] OR [All: mesothelioma*] OR [All: metasta*] OR [All:	17

Database	Search	Search String	Results
		"mycosis fungoid*"] OR [All: myelo*] OR [All: "myelodysplastic syndromes"] OR [All: neoplas*] OR [All: neuroblastoma*] OR [All: neuroma*] OR [All: nonmelanoma*] OR [All: nonseminoma*] OR [All: osteosarcoma*] OR [All: pancreatoblastoma*] OR [All: papillomatos*] OR [All: paraganglioma*] OR [All: paraneoplas*] OR [All: "persistent trophoblastic disease"] OR [All: phaeochromocytoma*] OR [All: pheochromocytoma*] OR [All: pineoblastoma*] OR [All: plasmacytoma*] OR [All: "polycythaemia vera"] OR [All: "pseudomyxoma peritonei"] OR [All: rhabdomyosarcoma*] OR [All: retinoblastoma*] OR [All: rhabdo* or sarcoma*] OR [All: schwannoma*] OR [All: seminoma*] OR [All: "sézary syndrome*"] OR [All: terato*] OR [All: thrombocythaemia*] OR [All: tumor*] OR [All: tumour*]] AND [[All: omop*] OR [All: "observational medical outcomes partnership"] OR [All: ohdsi] OR [All: "observational health data sciences and informatics"]] AND [E-Publication Date: (01/01/2014 TO 28/02/2025)]	
IEEE Xplore	S1	"All Metadata":cancer* OR "All Metadata":neoplas* OR "All Metadata":tumor* OR "All Metadata":tumour*) AND ("All Metadata":OMOP* OR "All Metadata": "Observational Medical Outcomes Partnership" OR "All Metadata":OHDSI OR "All Metadata":"Observational Health Data Sciences and Informatics" AND (Filters Applied: 01/01/2014 – 02/28/2025)	3
Google Scholar	#1	cancer OR neoplasm OR tumor OR tumour AND OMOP OR "Observational Medical Outcomes Partnership" OR OHDSI OR "Observational Health Data Sciences and Informatics" | Custom Range: 2014-2025, Sort by relevance, Search English pages	4450*

Notes:

The search strategies employed Medical Subject Headings (MeSH) terms for PubMed, CINAHL Subject Headings for CINAHL, Emtree terms for Embase, and related keywords to identify relevant literature discussing real-world implementations of cancer data transformation into OMOP CDM. For the IEEE Xplore database, the search strategy was slightly different due to a limitation on the usage of wildcards (restricted to only 10 per query).

*) Only the first 100 articles, sorted by relevance, were screened for the literature review.

Supplementary Table 8 | Inclusion and exclusion criteria for the literature search in both academic and gray literature

Criteria	Inclusion	Exclusion
"OMOP" or "OHDSI"	Mentioned "OMOP" or "OHDSI"	Did not mention "OMOP" or "OHDSI"
Cancer	Mentioned cancer-related data	Did not mention cancer-related data
Harmonization	Described a documented, step-by-step harmonization process of real-world cancer data from non-OMOP CDM sources (e.g., EHRs, cancer registries, claims data) into OMOP CDM.	Did not describe the harmonization process into OMOP CDM, but instead:<br>a. Discussed only vocabulary mapping without detailing the broader data transformation process<br>b. Focused on proposing the extension or development of OMOP CDM or OHDSI tools rather than data harmonization<br>c. Described data already in OMOP CDM format without detailing how it was transformed from its original source<br>d. Research was purely theoretical or conceptual (e.g., a proposal or planned harmonization effort without actual implementation)<br>e. The study used only synthetic datasets or simulated harmonization without real-world data
Full Text Accessibility	Full text was available and accessible for academic literature	Full text was unavailable or inaccessible for academic literature due to subscription or access restrictions, even after using both KI and SU student accounts, or was limited to an abstract-only format in conference proceedings
Language	Written in English	Written in languages other than English
Timeframe	Published between 1st January 2014 and 28th February 2025	Published before 1st January 2014

Supplementary Data 1 | Demonstration Scenario: Harmonizing Cancer Data from EHRs, National Cancer Registry Data, and Insurance Claims into OMOP CDM

A consortium of oncology research institutions received a large grant to harmonize EHRs data from multiple hospitals, the national cancer registry, and insurance claim data into OMOP CDM. This initiative aims to facilitate multi-site cancer research to analyze long-term treatment patterns and cost-effectiveness for lung cancer patients over the past decade.

Each hospital uses different data models, some employing openEHR, others based on HL7 FHIR. The EHRs contain structured data for diagnosis, some tumor characteristics, and treatment, and unstructured data with cancer staging are stored as unstructured text in clinical notes. The cancer registry contains structured data on diagnosis, tumor characteristics, and treatments. The claims data includes diagnosis codes, claimed treatment procedures and medication, and costs in structured data, but lacks direct clinical details such as tumor staging and standardized terminology. The claim database uses its own local system billing code.

To accommodate this harmonization, the institution forms a team comprising oncologists, surgeons, pharmacists, nurses, data engineers, data analysts, data managers from each data source, OMOP CDM experts, and health informaticians. The institution has no restrictions on open-source installations as long as it is with proper permissions and monitoring. Additionally, the required infrastructure is prepared and guaranteed to support this extensive harmonization initiative.

Demonstration of the Proposed Harmonization Process:

1. **Initiation**
   The first step, Initiation, focuses on laying the groundwork by assembling the team, defining the intended use case for harmonization, and familiarizing with the OHDSI ecosystem and related infrastructures.
   - ***Team Aspects***: An interdisciplinary team has been formed, covering all essential expertise required for data harmonization. The next step involves training to ensure all team members understand the harmonization process and are familiar with the OMOP CDM.
   - ***Infrastructure Aspects***: The institution has no restrictions on the installation of open-source tools, allowing the use of OHDSI tools and algorithms with appropriate permissions and monitoring. This step focuses on getting the team familiar with the OHDSI ecosystem.
   - ***Data Aspects***: The intended use case, inclusion criteria, key variables, and data sources have been defined. The objective is to analyze lung cancer patients from the past ten years to evaluate treatment patterns and cost-effectiveness, using a combination of EHRs, cancer registry, and claims data, all to be transformed into the OMOP CDM format.
     One remaining task is identifying similar projects, such as DIGICORE (DIGital Institute for Cancer Outcomes Research), a pan-EU research collaboration, and the iCAN Studyathon, which focuses on metastatic non-small cell lung cancer (mNSCLC) under the Research Council of Finland Flagship Programme. Aligning with such initiatives ensures consistent assumptions, supporting scalable evidence generation across projects

2. **Requirement Analysis**

   This phase focuses on assessing detailed requirements for the Team, Data, and Infrastructure Aspects to identify potential challenges or gaps and to develop appropriate mitigation strategies.

   - ***Team and Infrastructure Aspects***: No additional expertise is needed, and the infrastructure is confirmed to be sufficient to facilitate the harmonization process.
   - ***Data Aspects***: Data profiling is conducted using White Rabbit tools to analyze data information and syntactic structures. Additionally, the semantic vocabulary used and coverage are collected from data managers. The following details and potential issues are identified on each layer of consideration:
     - Data Information:
       - Data source types: EHRs, cancer registry, insurance claims database
       - Data content: Diagnosis (available in all data sources), tumor characteristics (available in cancer registry and EHRs), treatments (present across all sources, though treatment regimens in EHRs require derivation, possibly needing specialized tools), costs (available in claims database).
       - Data structure: Structured data (claims database and cancer registry); unstructured data (EHRs contain free-text entries in clinical notes for staging and biomarker data, necessitating text extraction techniques)
       - Data model adherence: openEHR and HL7 FHIR for EHRs, requiring specific tools to extract data from these models; no specific data model for others, in a SQL relational database.
       - Data quality: Potential issues identified include data duplication, inconsistent levels of granularity in cancer diagnoses across sources, and the complexity of merging datasets. Addressing these challenges requires careful deduplication, clinical validation with medical experts to ensure differing levels of detail represent the same clinical concepts, and the involvement of technical experts to design strategies that minimize information loss during integration.
     - Semantic Vocabulary: EHRs and cancer registry use ICD-10 for cancer diagnosis, ATC for medications, and SNOMED CT for other codes. Mapping ATC to RxNorm and ICD-10 to ICD-O-3 is necessary but relatively straightforward, as both vocabularies are supported within the OMOP CDM. SNOMED CT does not require additional mapping. In contrast, the claims database lacks standardized terminology and relies on billing codes, necessitating the use of a local dictionary and input from claims data managers during the mapping phase to avoid ambiguity and ensure accurate translation into standardized concepts.
     - Syntactic Structure: All sources follow a person-centric structure with clear relationships, and the required tables and fields have been identified.

Before progressing to the next phase, the analysis results are reviewed to ensure that key variables relevant to the research, such as diagnosis, tumor characteristics, treatment, and cost data, are validated by medical experts. Descriptive statistics are also generated to support the subsequent evaluation of the transformed data. Additionally, mitigation strategies are developed collaboratively with the team and thoroughly documented

3. **Design Plan**
   This step focuses on developing harmonization strategies, including evaluation and maintenance plans. In terms of Infrastructure Aspects, the infrastructure plan is established, and testing environments are set up to support unit testing and validation checks. In addition, all technologies chosen are ensured to be compatible with OMOP CDM and the institution's infrastructure and regulations.
   In terms of data aspects, transformation rules, including preprocessing and curation requirements identified in the previous steps, are also incorporated into the overall data harmonization strategy. For EHRs, preprocessing includes extracting data from openEHR and HL7 FHIR-based models using custom algorithms, extracting free-text information from clinical notes using NLP techniques, and applying ARTEMIS to derive treatment regimen information. This preprocessing is followed by curation and validation steps to ensure data accuracy and minimize information loss through iterative refinement.
   - Semantic Mapping: For EHRs and the cancer registry, Athena is used to help with mapping the source codes to OMOP standardized vocabularies. For the claims database, which lacks standardized terminology, Usagi is used for manual mapping, guided by a local data dictionary and support from the claims data manager to avoid ambiguity.
   - Syntactic Mapping: Once domain concepts are identified, they are mapped to the corresponding OMOP tables using Rabbit-In-a-Hat to document the ETL logic. Cancer trajectory episodes are mapped to the Episode table and linked to the Episode_event table

   Before advancing to the next phase, the mapping strategies are reviewed by the interdisciplinary team to ensure accuracy, consistency, and minimal information loss. The process is iteratively refined, and unit tests are developed to test the ETL logic.

4. **Technical Implementation**
   The technical implementation is carried out based on the designed plan using SQL. During this phase, all data from various sources is merged and deduplicated. After the transformation, evaluation is conducted, including mapping validation, data quality checks, and empirical model testing. This process is iterative and continues until all quality checks are successfully passed, ensuring that the data meets the required standards and is accurately represented in the OMOP CDM.

5. **Maintenance**
   Regular maintenance, managed by designated team members, is essential to support ongoing optimization efforts. This includes scheduled activities aimed at keeping both the data and supporting infrastructure up to date, as well as accommodating changes in data sources, tools, and technologies. Maintenance activities also involve addressing any misalignments or errors identified post-harmonization and proactively upgrading infrastructure or tools to meet future requirements. Continuous engagement with medical experts, data specialists, ETL developers, and OMOP CDM professionals is critical to ensure that the transformed data remains accurate, consistent, and valuable for ongoing research and analytics across diverse domains.

All steps are thoroughly documented, including decisions made, conventions followed, and any changes implemented, which provides a clear record for future revisits, harmonization efforts, and tracking changes over time.

Supplementary Data 2 | Interview Guide

Part A. Background & Experience

1. Could you tell me about your professional background and how it relates to cancer data and OMOP CDM? How long have you been using OMOP CDM?
2. What types of cancer data have you worked with (e.g., structured/unstructrued EHR data, pathology reports, genomic data, cancer registries, claims data, medical imaging, PROs)?
3. Could you describe your role and your experience in harmonizing cancer data into OMOP CDM? Which phases of the process have you been directly involved in (e.g., initiation, specification & profiling, data curation, ETL design, technical implementation, quality control, maintenance)?
4. Based on your experience, what are the key considerations when working with cancer data? Could you share the challenges you have encountered when harmonizing cancer data into OMOP CDM and what strategies or tools have you found most effective in addressing them?

Part B. Evaluation of Proposed Cancer Data Harmonization Process

1. Based on the demonstration and hypothetical scenarios, what are your initial impressions of the harmonization process? (General Perception)
2. From your perspective, how intuitive and practical is this process to implement? Are there any aspects that could make it easier to use? (Ease of Use)
3. Does the proposed process cover all essential aspects of cancer data harmonization? Are there any missing steps or areas needing improvement? (Completeness)
4. Do the steps follow a logical and efficient sequence? Are there any redundancies, gaps, or ways to optimize the workflow? (Efficiency)
5. How well does this process adapt to different cancer data sources (e.g., registries, EHRs, insurance claims)? Are there any contexts where it may not work effectively? (Generality)
6. How feasible is it to integrate this process into routine data harmonization workflows? What factors could facilitate or hinder its adoption? (Operationality)
7. What improvements would you suggest for enhancing the proposed harmonization process?
8. Is there anything else you would like to add regarding the proposed harmonization process's design or implementation?

Respondent Information

1. Sex
   a. Male
   b. Female
   c. Prefer not to say
2. Age Group (in years)
   a. 21-30
   b. 31-40
   c. 41-50
   d. 51-60
   e. >60
3. What is your role in the cancer data harmonization process?
   a. Academia & Research (e.g. research scientist, epidemiologist)
   b. Clinician or Medical Expert (e.g. doctor, nurse, pharmacist)
   c. Data Expert (e.g., health data owner, provider, steward)
   d. Extract-Transform-Load (ETL) Expert (e.g. data engineer, informatician, software developer)
   e. OMOP CDM Expert
   f. Other
4. How many years of experience do you have in transforming cancer data into OMOP CDM?
   a. 0-2 years
   b. 3-5 years
   c. 6-8 years
   d. 9-10 years
   e. >10 years

Cancer Data and Sources

5. What type(s) of cancer data sources have you harmonized into OMOP CDM?
   Refers to the original source of the data before transformation into OMOP CDM
   (Please select all that apply. If you select 'Other,' kindly specify the source.)
   a. Electronic Health Records (EHRs)
   b. Cancer Registries
   c. Biobanks
   d. Administrative & Claims Data
   e. Integrated Health or Research Databases
   f. Patient Reported Outcomes (PROs)
   g. Other
6. What type(s) of cancer-related data have you harmonized into OMOP CDM?
   Refers to the specific data elements transformed into OMOP CDM, regardless of source
   (Please select all that apply. If you select 'Other,' kindly specify the data type.)
   a. Cancer Diagnosis & Tumor Characteristics
   b. Genomic & Molecular Data
   c. Laboratory Data
   d. Imaging Data
   e. Medication & Treatment Data
   f. Other
7. What type(s) of data source structures have you harmonized into OMOP CDM? *(Please select all that apply)*
   a. Structured Data
   b. Semi-Structured Data
   c. Unstructured Data
8. Which data model(s) have you worked with from cancer data sources before harmonizing into OMOP CDM?
   a. HL7 FHIR (Fast Healthcare Interoperability Resources)
   b. openEHR
   c. No specific data model
   d. Other

Data Harmonization Process

9. What aspects of the source data do you always consider before data harmonization?
   (Please select all that apply. If you select 'Other,' kindly provide additional details.)
   a. Data type (EHRs, cancer registries, claims data, biobanks, genomic/molecular data)
   b. Data structure (structured, semi-structured, unstructured)
   c. Data completeness (e.g., missing values, duplicates)
   d. Vocabularies (e.g., local code, non-OMOP standardized vocabularies, required translation)
   e. Table structures & source attributes (e.g., field naming, relationships between tables, wide vs. long table format)
   f. Other
10. What step(s) do you (or your team) typically perform when harmonizing cancer data into OMOP CDM?
    Refers to the key tasks performed during data transformation process
    (Please select all that apply. If you select 'Other,' kindly provide additional details.)
    a. Define the use case for harmonization (e.g., establishing the aims and defining inclusion/exclusion criteria for research study)
    b. Set up OHDSI ecosystem (e.g., OMOP CDM, OHDSI tools, standardized vocabularies)
    c. Assemble an interdisciplinary group (e.g., medical expert, data expert, ETL expert, OMOP CDM expert)
    d. Analyze source data (e.g., assessing data information, vocabulary coverage, table structure and source attributes)
    e. Plan ETL process (e.g., designing how data will be extracted, transformed, and loaded into OMOP CDM)
    f. Perform data curation (e.g., cleaning, deduplication, de-identification, handling missing values, local code translation)
    g. Semantic mapping (e.g., mapping local/source codes to OMOP standardized vocabularies)
    h. Syntactic mapping (e.g., mapping source tables and data elements to OMOP CDM tables)
    i. Evaluation (e.g., quality control, running validation, data quality checks, benchmarking)
    j. Regular maintenance (e.g., regularly updating OMOP vocabularies, OHDSI tools, addressing data changes)
    k. Other
11. Which step(s) do you typically participate in during the harmonization process?
    Answers are the same as previous questions, but this question focuses on personal involvement
    (Please select all that apply. If you select 'Other,' kindly provide additional details.)
12. Which OHDSI open-source tools or algorithms have you used during the cancer data harmonization process into OMOP CDM?
    (Please select all that apply. If you select 'Other,' kindly provide additional details.)
    a. Achilles
    b. ARES
    c. Artemis
    d. Athena
    e. ATLAS
    f. Data Quality Dashboard
    g. KOIOS
    h. OncoRegimenFinder
    i. Rabbit In A Hat
    j. Usagi
    k. White Rabbit
    l. None (I have not used any OHDSI open-source tools or algorithms)
    m. Other
13. What challenges do you typically face during cancer data harmonization?
14. How do you usually address those challenges?

Evaluation of Proposed Cancer Data Harmonization Process into the OMOP CDM

15. After reviewing the proposed harmonization process, please evaluate the following statements based on your own direct experience with specific harmonization steps.
    Indicate your level of agreement using the provided Likert scale (Strongly Disagree, Disagree, Neutral, Agree, Strongly Agree). If a statement does not apply to your experience, please select "Unable to Answer."
    (Consider only the harmonization steps in which you have direct experience.)
    a. Based on my role and experience, I find the proposed harmonization steps are easy to follow
    b. The proposed harmonization process adequately covers all essential steps required to support my practical workflows and responsibilities
    c. The order and logical structure of the proposed harmonization steps align effectively with workflows I typically follow in my role
    d. The proposed harmonization process could be adaptable and generalizable to be applied across various cancer data types and sources, based on my opinion
16. What improvements would you suggest for enhancing the proposed harmonization process?
17. Are there specific considerations or additional steps you believe should be included in the process?
18. What potential challenges do you foresee when applying this process in practice?
19. Do you have any other feedback or comments?

Supplementary Fig. 3 | Questionnaire question list
